# Characteristics of hospital and health system initiatives to address social determinants of health in the United States: a scoping review of the peer-reviewed literature

**DOI:** 10.3389/fpubh.2024.1413205

**Published:** 2024-05-30

**Authors:** Pavani Rangachari, Alisha Thapa, Dawa Lhomu Sherpa, Keerthi Katukuri, Kashyap Ramadyani, Hiba Mohammed Jaidi, Lewis Goodrum

**Affiliations:** ^1^Department of Population Health and Leadership, School of Health Sciences, University of New Haven, West Haven, CT, United States; ^2^Northeast Medical Group, Yale New Haven Health System, Stratford, CT, United States

**Keywords:** social determinants of health, hospitals and health systems, health-related social needs, screening and referral, hot spotting, electronic health records, population health, public health

## Abstract

**Background:**

Despite the incentives and provisions created for hospitals by the US Affordable Care Act related to value-based payment and community health needs assessments, concerns remain regarding the adequacy and distribution of hospital efforts to address SDOH. This scoping review of the peer-reviewed literature identifies the key characteristics of hospital/health system initiatives to address SDOH in the US, to gain insight into the progress and gaps.

**Methods:**

PRISMA-ScR criteria were used to inform a scoping review of the literature. The article search was guided by an integrated framework of Healthy People SDOH domains and industry recommended SDOH types for hospitals. Three academic databases were searched for eligible articles from 1 January 2018 to 30 June 2023. Database searches yielded 3,027 articles, of which 70 peer-reviewed articles met the eligibility criteria for the review.

**Results:**

Most articles (73%) were published during or after 2020 and 37% were based in Northeast US. More initiatives were undertaken by academic health centers (34%) compared to safety-net facilities (16%). Most (79%) were research initiatives, including clinical trials (40%). Only 34% of all initiatives used the EHR to collect SDOH data. Most initiatives (73%) addressed two or more types of SDOH, e.g., food and housing. A majority (74%) were downstream initiatives to address individual health-related social needs (HRSNs). Only 9% were upstream efforts to address community-level structural SDOH, e.g., housing investments. Most initiatives (74%) involved hot spotting to target HRSNs of high-risk patients, while 26% relied on screening and referral. Most initiatives (60%) relied on internal capacity vs. community partnerships (4%). Health disparities received limited attention (11%). Challenges included implementation issues and limited evidence on the systemic impact and cost savings from interventions.

**Conclusion:**

Hospital/health system initiatives have predominantly taken the form of downstream initiatives to address HRSNs through hot-spotting or screening-and-referral. The emphasis on clinical trials coupled with lower use of EHR to collect SDOH data, limits transferability to safety-net facilities. Policymakers must create incentives for hospitals to invest in integrating SDOH data into EHR systems and harnessing community partnerships to address SDOH. Future research is needed on the systemic impact of hospital initiatives to address SDOH.

## Introduction

Social Determinants of Health (SDOH) refer to “the conditions in which people are born, grow, work, live, and age, and the wider set of forces and systems shaping the conditions of daily life” (1). Social Determinants have been found to account for considerably more variation in health and quality-of-life outcomes compared to clinical care (2, 3). Past research has estimated that over 900,000 deaths per year are attributable to social factors in the United States (4). An unequal distribution of SDOH at the community level is the root cause of health-related social needs (HRSNs) at the individual level (2). HRSNs refer to individuals’ social needs which in turn may include healthy foods, affordable housing, or transportation. For example, a given community may lack abundant affordable housing, however, local individuals may experience housing needs differently. In other words, HRSNs represent downstream manifestations of an unequal distribution of SDOH experienced by individuals. Distinguishing between SDOH and HRSNs is relevant for assessing the evidence and formulating effective responses at the policy and organizational levels (2, 5).

Nonprofit hospitals and health systems constitute nearly 50 % of all hospitals in the United States (US) and hold substantial financial resources (6, 7). There are 6,120 hospitals in the Unites States, including 5,129 community hospitals (84%) and 2,987 nonprofit community hospitals (49%). A total of 3,510 (57%) community hospitals belong to a health system (6), including multihospital systems and diversified single hospital systems. Nonprofit community hospitals are tax-exempt entities required by law to uplift the communities they serve. However, they have historically invested little in addressing SDOH (7, 8).

Although many policy initiatives have sought to encourage hospitals and health systems to address SDOH over the years, the Affordable Care Act (ACA) of 2010 is widely regarded as an inflection point in this regard. The ACA included provisions to shift payments to providers from fee-for-service to value-based care. Value-based payment models have created incentives for treating the whole patient across broad episodes of care over time, which in turn have challenged providers to focus beyond specific diseases to address unmet social needs, improve outcomes, and provide more value to patients and payers alike (8, 9). Beyond new payment incentives, the ACA also required nonprofit hospitals to conduct community health needs assessments (CHNAs) and develop community benefit implementation strategies every 3 years (10). These requirements in turn have shed light on a range of social conditions beyond traditional diseases, e.g., food insecurity, housing instability, that are of critical importance to individuals’ health and that hospitals had traditionally not addressed in a major way. In doing so, CHNAs have fostered increasing engagement among hospitals, health departments, and community-based organizations (9, 10).

### Study purpose and significance

This scoping review of peer-reviewed literature seeks to describe the characteristics of existing hospital and health system initiatives to address SDOH in the United States. This topic is significant for three reasons: (1) Persistent concerns related to the adequacy and distribution of hospital initiatives to address SDOH, (2) Recent environmental and policy influences that have served to accelerate hospital attention to SDOH, and (3) Wide variation in existing hospital and health system initiatives to address SDOH.

#### Persistent concerns related to the adequacy and distribution of hospital SDOH efforts

*First,* despite the incentives and provisions created for hospitals by the ACA related to value-based payment and CHNAs, concerns remain regarding the adequacy and distribution of hospital initiatives to address SDOH. For example, survey-based studies conducted during the COVID-19 pandemic showed that rural, critical access, and safety-net hospitals screened for a similar number of social needs as non-safety net hospitals but implemented fewer interventions and reported fewer community partnerships, indicating that vulnerable populations may have benefitted the least from hospital efforts to address SDOH during the pandemic (11, 12).

#### Recent environmental influences that have served to accelerate hospital attention to SDOH

*Second,* beyond the incentives and provisions created by the ACA for hospitals to address SDOH, broader environmental and policy influences have paved the way for heightened attention to SDOH by hospitals in the US. For example, COVID-19 served to both expose and exacerbate the racial/ethnic disparities in health and healthcare, with African American and Hispanic populations experiencing disproportionately higher rates of SARS-CoV-2 infection, hospitalization, and mortality compared to non-Hispanic White populations during the pandemic (2, 11). Consequently, for the first time in recent US history, the Department of Health and Human Services (HHS), and all other federal agencies, have been charged with a whole-of-government, multi-sector approach to identify and address the structural barriers to achieving health equity (2, 13). Aligned with this emphasis, Healthy People 2030, an HHS initiative to address the nation’s public health priorities, has incorporated an overarching goal specific to SDOH: “Create social, physical, and economic environments that promote attaining the full potential for health and well-being for all” (14). The benchmarks set by Healthy People in turn are expected to enable the HHS to monitor progress and motivate and focus action related to improving SDOH. A pivotal federal agency under HHS’ oversight is the Centers for Medicare and Medicaid Services (CMS). CMS provides health coverage to more than 160 million people through Medicare, Medicaid, the Children’s Health Insurance Program, and the Health Insurance Marketplace (15). Correspondingly, CMS is the single largest payer for healthcare services in the US and thus would play a critical role in driving improvements in quality, equity, and outcomes in the healthcare system over the next decade. Correspondingly, the US HHS health equity initiative may be viewed as an urgent call to action for the nation’s hospitals and health systems.

Other recent mile markers signaling healthcare’s accelerating attention to social determinants include the adoption of Z codes for documenting social risk factors in the International Classification of Diseases, Tenth Revision (ICD-10) (16), the American Academy of Pediatrics’ endorsement of universal screening for food insecurity (17), the recent policy statement on social needs from the American College of Physicians (18), and a recent initiative by the American Hospital Association to “Redefine the H,” a campaign that seeks to associate the “H” sign for a nearby hospital with communitywide efforts to address health more broadly (19). Moreover, in recognition of the burgeoning literature in the field, the National Library of Medicine (NLM) has made Social Determinants of Health a Medical Subject Headings (MeSH) term to enable literature searches on this topic (20). Finally, the growing evidence of the success of value-based care models in addressing the whole person’s (clinical and social) needs has also served to bolster attention to SDOH by hospitals and health systems (8).

#### Wide variations in existing hospital and health system initiatives to address SDOH

*Third,* wide variations have been reported in the emphasis and scope of existing hospital and health system initiatives to address SDOH in the US. For example, a common approach has been to undertake “downstream” initiatives to address health-related social needs HRSNs at the individual level. These types of interventions often involve screening and referral, i.e., screening patients for social needs and referring those in need to community-based resources (9, 21). There may be variability even among downstream initiatives undertaken by hospitals and health systems. Some interventions may involve hot spotting to address HRSNs of high-risk patients, while others may focus on screening and referral (22). Also, some interventions may focus on addressing multiple social needs (healthcare access, food, transportation), while others may target a single social need (housing) (9, 21). By comparison, it may also be possible for hospitals to undertake “upstream” initiatives to address structural SDOH in their communities. For example, in the context of housing, nonprofit hospitals could invest in improving the quality of existing housing stock within the community, building up the supply of quality, affordable housing, and advocating for federal and state policies that strengthen and stabilize housing (7).

In the context of the three forementioned reasons, this scoping review is timely and significant in describing the key characteristics of hospital and health system initiatives to address SDOH in the US, to gain insight into the progress and gaps and identify implications for practice, policy, and research. Notably this scoping review of the peer-reviewed literature seeks to understand “what” hospitals and health systems are doing to address SDOH in the US, to address broader questions related to the adequacy and distribution of SDOH initiatives. By the same token, the question of “how effectively” hospitals are addressing SDOH, to address broader questions related to the effectiveness and outcomes impact of SDOH initiatives, is beyond the scope of this paper. By examining the distribution of SDOH initiatives by safety-net hospital status, by US geographic region; and characterizing the emphasis of hospital initiatives with regard to addressing downstream HRSNs vs. upstream SDOH; their reliance on internal capacity vs. community partnerships to provide services, and so on, this paper seeks to shed light on the progress and gaps with respect to the adequacy and distribution of hospital and health system initiatives to address SDOH, to identify meaningful implications for practice, policy, and future research. Correspondingly, the primary purpose of this paper is to describe the key characteristics of hospital and health system initiatives to address SDOH in the US. Questions of effectiveness and impact on outcomes of hospital SDOH initiatives will be addressed by the authors in future publications.

## Literature review

Despite growing attention to SDOH and the role of the healthcare sector in addressing them, there are no available scoping reviews of the peer-reviewed literature on hospital and health system-led initiatives to address SDOH. Existing reviews on the topic have focused on specific subtopics of SDOH (e.g., reviews of hospital partnerships to promote population health) (23), specific SDOH (e.g., health system efforts to address housing) (24), specific outcomes (e.g., impact of social support on hospital readmission rates) (25), specific diseases (e.g., impact of social determinants in spine surgery) (26), or specific populations (e.g., social risks among primary care patients) (27).

It is noteworthy that a systematic review of safety net health centers’ efforts to address SDOH was recently undertaken in 2022 (28). However, that review was specific to Health Resources Service Administration (HRSA)-funded federally qualified health centers. It did not address the traditional community hospital’s role in addressing SDOH. Moreover, the review questions were focused on examining downstream initiatives to address HRSNs undertaken by HRSA-funded health centers, specifically, their effectiveness in integrating screening and referral for HRSNs into care practices, using the Electronic Health Record (EHR) (28). By comparison, this review’s scope is broader in seeking to capture both downstream and upstream initiatives to address SDOH, on the part of hospitals and health systems. While there are nearly 1,400 HRSA-funded health centers in the US (28), there are over 5,000 community hospitals in the US, including over 3,500 that belong to a system (6). Therefore, a gap exists in having a more comprehensive characterization of efforts being undertaken by hospitals and health systems to address SDOH in the US. This review seeks to address this gap.

This scoping review also makes conceptual contributions to the literature in two areas: (1) What types of SDOH do hospitals and health systems need to address; and (2) How to characterize hospital and health system efforts to address SDOH. Regarding #1, *types of SDOH hospitals/health systems need to address*, this review integrates the Healthy People SDOH framework with the Institute for Healthcare Improvement (IHI) listing of SDOH types to inform the literature search for hospital initiatives to address SDOH (29–31). Healthy People 2030 provides a framework for organizing SDOH into five areas: (1) Economic stability; (2) Education access and quality; (3) Social and community context; (4) Healthcare access and quality; and (5) Neighborhood and built environment. Within each domain, Healthy People outlines individual SDOH factors (29). As a creation of the US federal agency housing the CMS, Healthy People has gained increasing popularity in informing hospital-led initiatives to address SDOH. For example, Pourat et al. (28) use Healthy People to guide their systematic review of efforts undertaken by HRSA-funded health centers to address SDOH.

**Figure 1:**
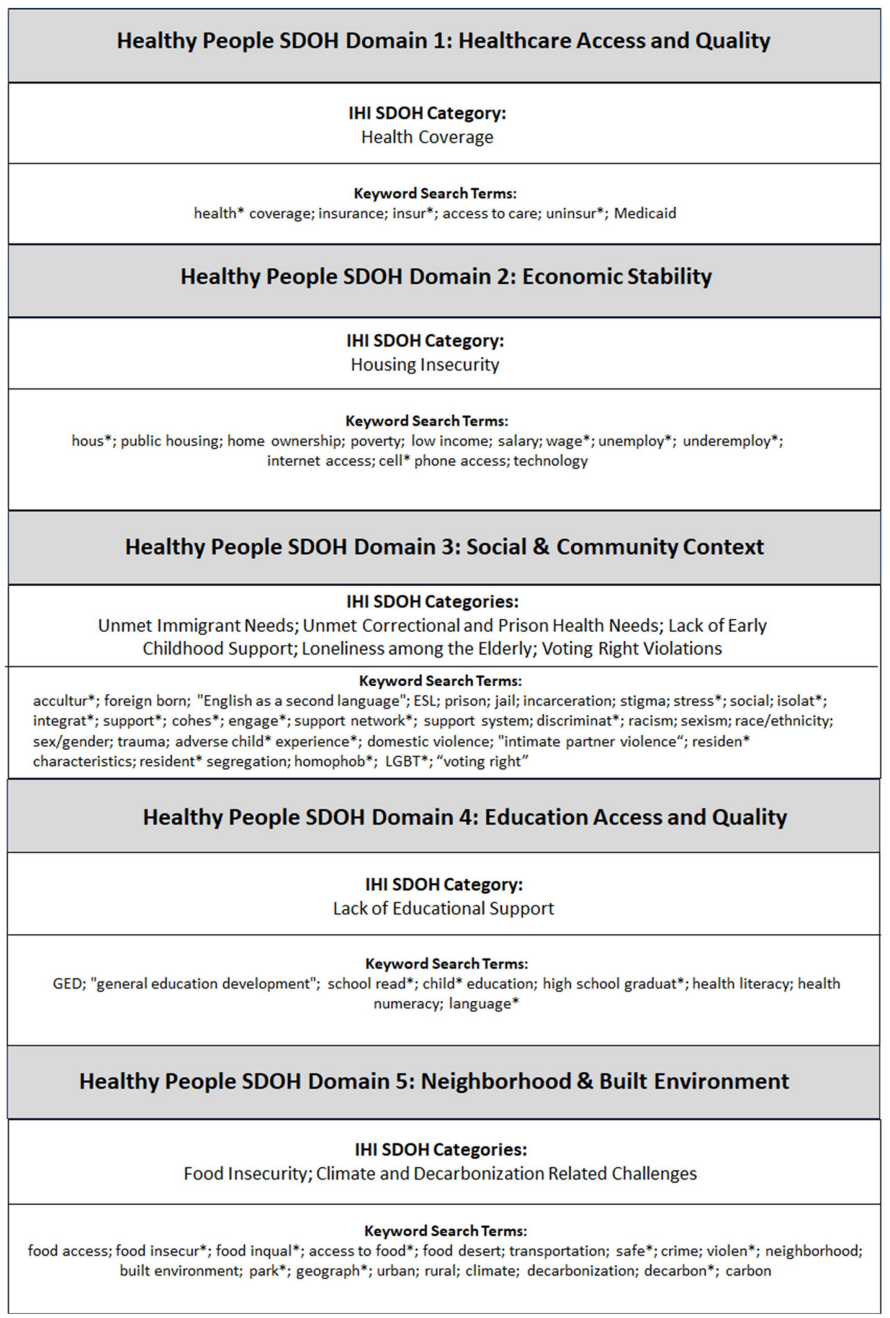
Integrated framework to inform the article search strategy.

However, the individual SDOH factors specified under Healthy People domains may not adequately encompass the types of SDOH hospitals should focus on. To capture the latter, it would be important to pay attention to the voice of the healthcare industry. In 2021, the venerable IHI called upon hospital leaders to proactively address 10 types of SDOH within their communities: (1) Health coverage, (2) Food insecurity, (3) Housing instability, (4) Unmet immigrant needs, (5) Unmet correctional health needs, (6) Climate and decarbonization related challenges, (7) Voting right violations, (8) Lack of educational support, (9) Lack of early childhood support, and (10) Lack of social support among the older adults (30, 31). While several of the IHI SDOH types are already represented on the Healthy People list (e.g., housing and food insecurity), there are other SDOH types on the IHI listing (e.g., climate and decarbonization, voting right violations, unmet immigrant needs, and lack of social support among the older adults) that are not on the Healthy People list. To address these inconsistencies and to ensure that the voice of the healthcare industry is represented, this review integrates Healthy People with the IHI listing to develop a comprehensive framework for determining the types of SDOH hospitals need to address, which in turn provides the foundation for the article search for this scoping review (see Figure 1).

Regarding #2, i.e., *how to characterize hospital and health system efforts to address SDOH*, the existing literature is limited to frameworks for characterizing hospital efforts to integrate SDOH data into hospital care practices through use of Electronic Health Records (EHR). For example, DeVoe et al. (32) and Cottrell et al. (33) have identified four steps that hospitals would need to follow to accomplish this, and Pourat et al. (28) in turn have leveraged these frameworks in their systematic review of HRSA-funded health center efforts to address SDOH. The focus of existing frameworks indicates an emphasis on downstream initiatives to address HRSNs. In other words, existing frameworks do not account for upstream initiatives that could be undertaken by hospitals and health systems to address structural SDOH at the community level (7, 24). Moreover, existing frameworks miss the variation that exists in hospital-led downstream interventions to address SDOH, including initiatives that may be independent of EHR use (34). This suggests the need for a broader framework to characterize hospital and health system initiatives to address SDOH. This review addresses this gap by capturing the full spectrum of hospital and health system initiatives to address SDOH described in the peer reviewed literature, while also leveraging existing frameworks to characterize use of EHR technology to address SDOH.

### Review questions

The objective of this review is to describe the characteristics of existing hospital and health system initiatives to address SDOH in the US. The specific review questions are outlined below.

1. RQ#1: What are the article characteristics of hospital and health system initiatives to address SDOH in the US, by publication year, article type, methodology, and inclusion of outcome measures?
2. RQ#2: What are the descriptive characteristics of hospital and health system initiatives to address SDOH in the US, e.g., by the number and type of SDOH addressed, type of hospital organization, US geographic region, conditions targeted, and services provided?
3. RQ#3: What is the emphasis of hospital/health system initiatives to address SDOH with respect to the following characteristics?

- *Addressing downstream HRSNs vs upstream SDOH.*
- *Screening and referral* vs. *hot spotting.*
- *Quality improvement* vs. *population health management.*
- *Reliance on internal capacity* vs. *community partnerships to provide services.*
- *Reducing health disparities and/or promoting health equity.*

1. RQ#4: Have hospitals and health systems utilized the Electronic Health Record (EHR) to integrate SDOH data into care practices?
2. RQ#5: What major challenges have been reported in hospital and health system initiatives to address SDOH?

### Rationale for a scoping review

According to Sucharew and Macaluso (35), scoping reviews can be useful for answering broad questions, such as “What information has been presented on this topic in the literature?” which in turn is fully consistent with what this review seeks to accomplish. To-date, there have been no comprehensive reviews of peer-reviewed literature to obtain the *scope of knowledge on characteristics of hospital and health system-led initiatives to address SDOH in the US*. This paper seeks to address this gap, and the review questions in turn, are aligned with this purpose.

## Methodology

PRISMA-ScR criteria were used to inform a scoping review of the literature (36). Three academic databases were searched for eligible sources of evidence from 1 January 2018 through 30 June 2023. The review protocol (Supplementary Data Sheet 1) was developed based on guidelines for scoping reviews provided by the Joanna Briggs Institute (JBI) (37). The protocol was not registered. The PRISMA-ScR checklist is included in Supplementary Data Sheet 2.

### Information sources

This scoping review sought to identify published research articles, review articles, and case reports to address the review questions. Three academic databases, PubMed, ABI/Inform (Pro Quest), and Academic Search Premier were searched for eligible articles over a 5+ year timeframe between 1 January 2018 and 30 June 2023. Since COVID-19 is known to have played a significant role in elevating the topic of SDOH to center stage in health policy debates (2, 11), papers published over the period spanning 2 years pre/post the pandemic year (2020) were considered for inclusion. The article search was conducted in September 2023. Given the topic of interest, the three forementioned academic databases were selected to ensure maximum coverage across biomedical science, clinical practice, social science, and health policy domains.

### Search strategy

As mentioned earlier, this paper integrates the Healthy People 2030 SDOH framework and the IHI SDOH types to inform the search strategy (Figure 1). To identify individual SDOH search terms under the Healthy People SDOH domains, this study leveraged standardized SDOH terminology and keywords representing specific social and behavioral determinants of health in Healthy People, as well as published search algorithms from existing reviews that utilized the Healthy People framework (28, 38). These terms were then supplemented with individual search terms to capture the IHI SDOH types (31).

Next, Boolean search operators were used to combine all individual SDOH terms with a set of search terms to capture the care setting of interest. As mentioned earlier, 57% of community hospitals in the US belong to health systems. In addition, over 220 nonprofit community hospitals are classified as academic medical or health centers (6). Importantly, as indicated earlier, surveys conducted during COVID-19 have found that safety-net hospitals are engaging in fewer strategies to address the SDOH compared to non-safety net hospitals. Given that this review seeks to address broader questions of adequacy and distribution hospital initiatives to address SDOH, it would be important to know if this disparity between safety-net and non-safety-net hospitals is reflected in the peer-reviewed literature on hospital initiatives to address SDOH in the pandemic/post-pandemic period. In the US, safety-net hospitals could include Safety Net Health Centers (SNHCs), Community Health Centers (CHCs), Federally Qualified Health Centers (FQHCs), or Minority-Serving Hospitals (MSHs). Historically, the terminology has sought to distinguish safety-net facilities serving low-income, vulnerable populations supported by social welfare payment sources. The variety of terms used to capture the hospital care setting in the US healthcare industry in turn necessitated casting a wide net to capture the hospital care setting for this review. In essence, the search terms of “hospital,” “health system,” “health center,” and “medical center,” were all deemed to be relevant for capturing the care setting of interest for this scoping review. Finally, yet importantly, the forementioned two sets of search terms, i.e., terms used to capture (1) individual SDOH factors and (2) the hospital care setting, were combined with (3) NLM MeSH terms (Medical Subject Headings) to capture the concept of social determinants of health. The full electronic search strategy used on PubMed is included as an example, in Supplementary Data Sheet 3.

### Eligibility criteria

The following were the eligibility criteria for article inclusion in this scoping review:

1. The article must be published within the date range 1 January 2018 through 30 June 2023.
2. The article must be published in English language.
3. The article must be published in a peer-reviewed journal. (The following sources were excluded: conference papers, working papers, wire feeds, reports, abstracts, books, trade journals, dissertations, theses, and magazines and other non-scholarly sources.)
4. The article must be based in the United States. (One or more of the authors’ affiliations need to be based in the US, and the article needs to describe a hospital or health system-led initiative to address SDOH that was based in the US.)
5. The article must be of an eligible article type, i.e., (i) research article, (ii) review article, or (iii) case report. Research articles considered for inclusion were based on empirical data collected from clinical trials, cohort studies, case–control studies, secondary data analysis, cross-sectional studies, and other types of quantitative, qualitative, and mixed-method studies. Also considered for inclusion were case reports, systematic reviews, and scoping reviews. (The following article types were excluded: study protocols, editorial articles, opinion papers and discussion papers.)
6. The article must be available in full text either through an open source or the original publishing source.
7. The article must be within the study scope. The criteria used for determination of review scope are described below.
8. The article must meet critical appraisal criteria outlined by the JBI (37) or the Mixed-Method Appraisal Tool (MMAT) (39), whichever is applicable.

As described in the article selection process (below), the forementioned set of eligibility criteria was applied over three of the four stages of the article selection process, including (1) restrictions applied during academic database search, (2) article screening, and (3) full-text review. Following full-text review, (4) selected articles that met the critical appraisal criteria were included in the review.

To be considered within the study scope (eligibility criterion #7 above), the article needed to describe a hospital or health system-led initiative based in the US to address SDOH at the patient or community level. By comparison, articles determined to be outside the scope of the study included research initiatives based on literature reviews or secondary analysis of national, statewide, or regional databases, to describe broad socioeconomic challenges and put forth strategies, policies, and programs for addressing them. Examples include a systematic review of technologies for prehospital communication and coordination (40); analysis of the national cardiovascular data registry to examine neighborhood socioeconomic disadvantage and care after myocardial infarction (41); and cross-sectional validation of the PROMIS-Preference scoring system by its association with SDOH (42). It would be relevant to note that although review articles were considered for inclusion, most reviews were deemed to be outside the scope, since they pertained to the broader impact of SDOH on specific conditions (e.g., diabetes) or outcomes (readmissions), often assessed at a national level. Like reviews, most analytic studies were multi-year, state, or national-level analyses of SDOH that were determined to be outside the study scope. By comparison, most clinical trials were included since they pertained to hospital or health system-led initiatives to address SDOH.

### Process for selecting sources of evidence

The three academic databases searched were equipped with search capabilities that enabled the exclusion of articles based on many of the eligibility criteria, even prior to downloading the articles for duplicate removal and title and abstract screening. This included the exclusion of articles published outside of the selected date range for this study; articles that were not published in peer-reviewed journals; articles that were not in English language; articles that were not based in the US; articles that were not of an eligible article type; and articles that were not available in full text either from an open source or the original publishing source.

Following the database search, all articles identified were collated, and uploaded into a reference management system (Zotero 5.0) for the removal of duplicates. After duplicate removal, article titles and abstracts were screened for potential inclusion, based on the eligibility criteria. Lastly, articles identified for inclusion were retrieved in full text for further review. Articles selected for inclusion following full-text review were subjected to critical appraisal using JBI checklists (for clinical trials, cross-sectional studies, qualitative studies, and review articles) (37). The MMAT was used to appraise mixed-method studies (39). Only articles that met the critical appraisal criteria were identified for final inclusion in the scoping review. At each stage, we resolved conflicts in inclusion or exclusion by discussion and achieving consensus among three independent reviewers who in turn were supported by two coding assistants.

### Process for charting data items for individual sources of evidence

All included articles were reviewed to retrieve and chart five categories of data (Data Categories 1–5) to address the corresponding five research questions. Supplementary Data Sheet 4, Charts 1–4 include all data collected/charted on individual sources of evidence.

Data Category #1: This includes data items for characterizing article characteristics on hospital and health system initiatives to address SDOH, aligned with RQ#1, including the publication year, article type, methodology, and inclusion of outcome measures.
Data Category #2: This includes data items for capturing the descriptive characteristics of hospital initiatives to address SDOH, aligned with RQ #2, including the number and type of SDOH addressed, type of hospital organization, US geographic region, targeted diseases/conditions, and services offered as part of the initiative.
Data Category #3: This includes data items for capturing additional characteristics of hospital initiatives to address SDOH, aligned with RQ #3, i.e., the emphasis on: (1) upstream vs. downstream SDOH; (2) screening and referral vs. hot spotting, (3) quality improvement vs. population health management, (4) reliance on internal capacity vs. community partnerships to provide services; and (5) reducing health disparities and/or promoting health equity.
First, as discussed earlier, hospital and health systems initiatives could take the form of downstream initiatives to address HRSN at the individual level or upstream initiatives to address structural SDOH at the community level. Second, hospital and health system initiatives could take the form of (1) screening and referral to address unmet social needs; or (2) a hot spotting approach to target a defined population at-risk, be it patients who visit the facility, e.g., chronic disease patients, or high utilizers within the community. Third, hospital and health system initiatives could be designed as Quality Improvement (QI) initiatives or Population Health Management (PHM) initiatives or a mix of QI and PHM. The focus of QI is on enhancing processes within the organization to deliver higher quality care to individual patients, while PHM focuses on improving the health outcomes of a defined population by reducing social risk. PHM involves analyzing data to understand health outcomes, trends, and disparities within the population, implementing interventions, and measuring impact. Fourth, hospitals could rely on built internal capacity (e.g., hospital-based food pantries) or rely on community partnerships to provide services (e.g., partnership with a community farm to offer healthy foods). Fifth, hospital and health system SDOH initiatives may be designed to have special emphasis on reducing health disparities and/or improving health equity which in turn is captured based on whether the initiative incorporates specific measures of health disparities or devotes specific discussion points to health disparities or health equity.
Data Category #4: This includes data items to capture hospital adherence to an existing four-step framework for using the EHR to integrate SDOH into care practices, in alignment with RQ#4 (32, 33). Supplementary Data Sheet 4, Chart 3 summarizes adherence to each of the four steps with a yes/no measure, while Supplementary Data Sheet 5 (raw dataset) includes a more detailed assessment of adherence to various sub steps within each of these four steps (33).
Data Category #5: This includes data items to capture the challenges encountered by hospitals and health systems in addressing SDOH, in alignment with RQ#5. The data items described under all five data categories were charted in multiple spreadsheet templates included in Supplementary Data Sheet 5, the raw dataset for the study.

### Process for synthesizing results

The data collected and charted from articles were summarized using counts, aggregates, and proportions for analysis and interpretation based on the review questions. This process helped to synthesize the evidence related to the progress and gaps in hospital initiatives to address SDOH as well as the state of the science pertaining to hospital initiatives to address SDOH.

## Results

### Selection of sources of evidence

**Figure 2:**
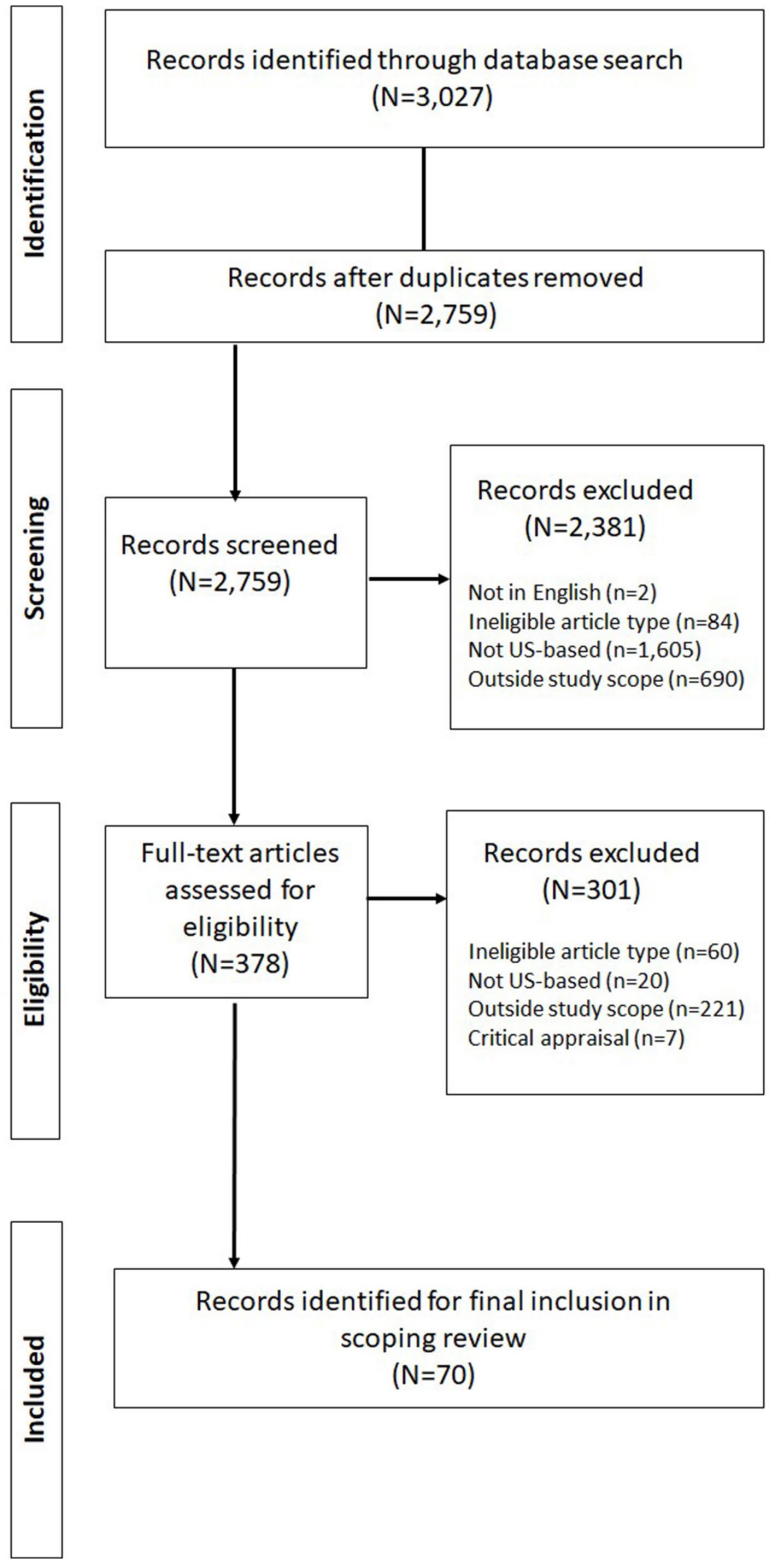
PRISMA flow chart for article selection.

A total of 3,027 articles were identified from the database search for downloading to the Zotero reference management system for duplicate removal and screening. Notably, the initial search with just the date restriction yielded a total of 21,246 records from the three databases (including 8,772 from PubMed, 10,436 on ABI/Inform, and 2,038 on Academic Search Premier). Of these, 18,219 were removed by applying additional database restrictions based on the exclusion criteria, i.e., not being in English (718), not being from a peer-reviewed source (9,607), not being an eligible article type (2,928), and non-availability of full text from an open source or the original source (4,966). Of the total of 3,027 articles downloaded, 268 duplicates were removed, while the remaining 2,759 progressed to title and abstract screening. After screening, a total of 2,381 articles were excluded for various reasons, including, 2 for not being in English, 84 for not being an eligible article type, 1,605 for not being based in the US, and 690 for being outside the scope of the study. The remaining 378 articles were retrieved for full text review following which, 60 were excluded for not being an eligible article type, 20 for not being based in the US, and 221 for being outside the scope of the study. The remaining 77 articles were subjected to critical appraisal, following which 7 were excluded, leaving a total of 70 articles for final inclusion in the scoping review (23, 24, 27, 34, 43–108) The search results are summarized on the PRISMA chart in Figure 2.

### Characteristics of sources of evidence

Supplementary Data Sheet 4, Chart 1 includes article characteristics associated with each of the 70 included articles (individual sources of evidence), corresponding to RQ#1.

### Results of individual sources of evidence

Supplementary Data Sheet 4, Charts 2–4 together, chart the results corresponding to the remaining review questions (RQ#2 through RQ#5) for all 70 articles.

### Synthesis of results

#### Results based on article characteristics

**Figure 3:**
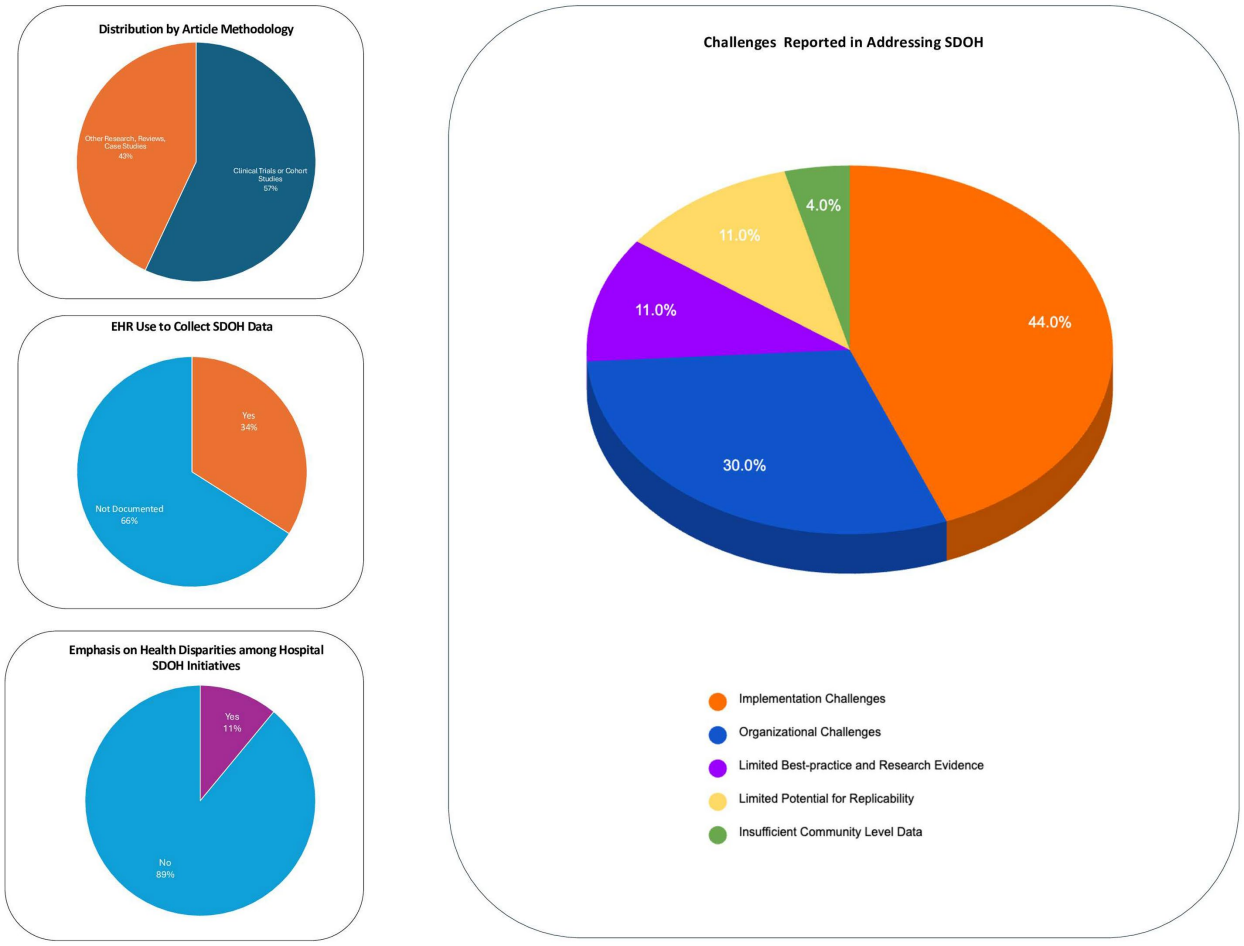
Methodology, EHR Use, Emphasis on Health Disparities, Challenges Reported.

Table 1 summarizes key article characteristics in response to RQ #1. Results show that most were research articles (79%), with a majority (57%) being either Randomized Clinical Trials (RCTs) (40%) or cohort studies (17%). Figure 3 (top left) provides a graph of the breakdown by article methodology. By year of publication, most (73%) were published in the pandemic period (2020 or later). Also, 90% of the articles (63/70) included outcome measures for tracking success of the initiative, be they clinical outcome measures (e.g., viral suppression among HIV patients) which were included in 67% of the articles and/or social outcome measures (e.g., affordable housing placements) which were included in 50% of the articles.

**Table 1:** Article characteristics.

Characteristics	Frequency	Percentage
**Publication year (*N* = 70)**		
2018	5	7.10%
2019	14	20.0%
2020	16	22.86%
2021	13	18.57%
2022	15	21.43%
2023	7	10.0%
Total	70	100%
**Article type (*N* = 70)**		
Research	55	78.57%
Review	4	5.70%
Case study/report	11	15.71%
Total	70	100%
**Article methodology (*N* = 70)**		
Randomized controlled trials	28	40.00%
Quasi-experimental study	2	2.86%
Observational cohort study	12	17.14%
Analytical cross-sectional or time-series study	9	12.85%
Case study/report	11	15.71%
Qualitative research	4	5.71%
Review	4	5.70%
Total	70	100%
**Inclusion of any outcome measures (*N* = 70)**		
Yes	63	90.0%
No	7	10.0%
Total	70	100%
**Inclusion of clinical outcome measures (*N* = 70)**		
Yes	47	67.14%
No	23	32.86%
Total	70	100%
**Inclusion of social outcome measures (*N* = 70)**		
Yes	35	50.0%
No	35	50.0%
Total	70	100%

#### Results based on review questions

**Figure 4:**
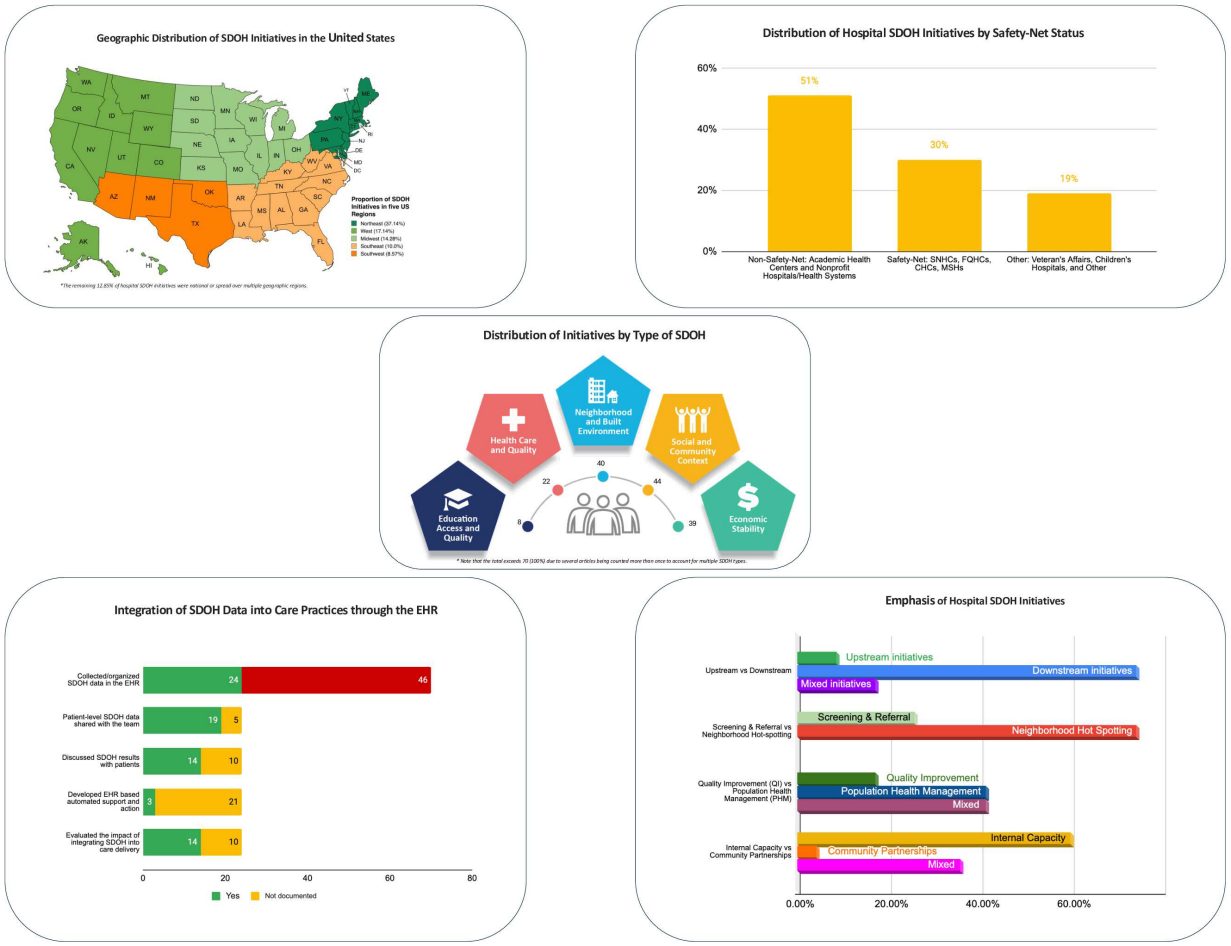
Characteristics of hospital initiatives to address SDOH.

Table 2 summarizes descriptive characteristics in response to RQ#2. By type of hospital organization, a combined 51% of the initiatives were undertaken by non-safety-net hospitals, including Academic Health Centers (34%) or Nonprofit Community Hospitals/Health Systems (17%). By comparison, a combined 30% of the initiatives were accounted for by safety-net hospitals, including Safety-Net Health Centers (16%), Federally Qualified Health Centers (10%), Community Health Centers (3%), and Minority Serving Hospitals (1%). Figure 4 (top right) provides a graphical depiction of the comparison between non-safety net and safety net hospitals. The results by US geographic region indicate that 37% (26) of the initiatives were based in the Northeast US, 17% (12) in the West coast, and 14% (10) in the Midwest. Figure 4 (top left) includes a map of the US depicting the regional distribution of hospital SDOH initiatives.

**Table 2:** Descriptive characteristics of hospital and health system initiatives to address SDOH.

Characteristics	Frequency	Percentage
**Number of SDOH targeted (*N* = 70 articles)**		
Single	16	22.86%
Multiple	51	72.86%
Not specified	3	4.28%
Total	70	100%
**Types of SDOH targeted (*N* = 70 articles)**		
Social and community context	44	62.86%
Neighborhood and built environment	40	57.14%
Economic stability	39	55.71%
Healthcare access and quality	22	31.43%
Education access and quality	8	11.43%
Not specified	3	4.28%
Total	156^*^	222.85%^*^
**Type of hospital organization (*N* = 70 articles)**		
Academic health center	24	34.29%
Nonprofit community hospital/health system	12	17.14%
Safety-net health center	11	15.71%
Community health center	2	2.9%
Minority serving hospital	1	1.43%
Federally qualified health center	7	10.0%
Veteran affairs health system	6	8.57%
Children’s hospital	4	5.71%
Other	3	4.28%
Total	70	100%
**Geographic region of the United States (*N* = 70 articles)**		
Northeast	26	37.14%
Southeast	7	10.0%
West	12	17.14%
Southwest	6	8.57%
Midwest	10	14.28%
National	3	4.28%
Multiple geographic regions	3	4.28%
Not specified	3	4.28%
Total	70	100%
**Diseases, conditions, outcomes addressed (*N* = 70 articles)**		
Chronic and infectious illness	23	32.86%
Substance use and lifestyle	7	10.0%
Primary care utilization	2	2.86%
High-risk healthcare utilization including readmission risk	13	18.57%
No shows	1	1.43%
Social needs	8	11.43%
Sexual assault survivorship	1	1.43%
Not specified/not applicable	15	21.43%
Total	70	100%
**Services provided (*N* = 70 articles)**		
Community health worker services	24	34.28%
Screening and referral for social needs	15	21.43%
Financial support services	6	8.57%
Digital health interventions	4	5.71%
Other initiatives	16	22.86%
Not documented	5	7.14%
Total	70	100%

^*^Note that the total exceeds 70, due to several articles being counted more than once to account for multiple types of SDOH addressed.

Results also revealed that the majority 51 (73%) of the initiatives addressed two or more (multiple) SDOH concurrently. The most frequently addressed health-related social needs were food, housing, education, transportation, and financial security. Figure 4 (center) includes a pictorial depiction of the distribution of initiatives by Healthy People Domain. As indicated, most initiatives fell under Social and Community Context (44), followed by Neighborhood and Built Environment (40), Economic Stability (39), Healthcare Access and Quality (22), and Education Access and Quality (8). Notably, this total exceeds the total number of articles (70), since several articles were counted more than once to account for multiple SDOH addressed by each initiative. By targeted diseases/conditions, chronic diseases received the most attention (33%); followed by high-risk for healthcare use including readmission (19%). By services offered, 34% of the initiatives offered community health workers, including navigation, coordination, and self-management support; 21% offered screening and referral for social needs; 9% offered financial support, while 6% offered digital health services, e.g., telemedicine.

Table 3 presents results related to RQ #3 (i.e., additional characteristics describing the emphasis of hospital initiatives to address SDOH), and Figure 4 (bottom right) provides a graphical depiction of the key results related to RQ#3. As indicated, most hospital/health system initiatives 74% (52) were downstream efforts to address health related social needs at the individual level either through hot spotting (targeting high-risk patients in the hospital or community) or through screening and referral of patients for HRSNs. Only 9% (6) were upstream efforts to address structural SDOH at a community level. The remaining 17% (12) were mixed in having both downstream and upstream elements.

**Table 3:** Additional characteristics of hospital initiatives to address SDOH.

Characteristics	Frequency	Percentage
**Upstream initiative vs. downstream initiative to address SDOH (*N* = 70)**		
Downstream initiatives	52	74.28%
Upstream initiatives	6	8.57%
Mixed initiatives	12	17.14%
Total	70	100%
**Screening and Referral vs. hot spotting (*N* = 70)**		
Hot spotting	52	74.29%
Screening and referral	18	25.71%
Total	70	100%
**Quality improvement (QI) vs. population health management (PHM) (*N* = 70)**		
Population health management	29	41.43%
Quality improvement	12	17.14%
Mixed	29	41.43%
Total	70	100%
**Internal capacity vs. community partnerships to address SDOH (*N* = 70)**		
Internal capacity	42	60.0%
Community partnerships	3	4.28%%
Mixed	25	35.71%
Total	70	100%
**Initiatives focused on reducing health disparities and/or improving health equity (*N* = 70)**		
Yes	8	11.43%
No/not documented	62	88.57%
Total	70	100%

Upstream initiatives took a variety of forms: (i) direct investments in community programs; (ii) policy advocacy to promote population health; and (iii) external or community partnerships to address HRSNs and promote population health. One review of the gray literature (86) found that between 2017 to 2019, 57 health systems (collectively, 917 hospitals), together made direct investments in 78 unique community-based programs to the tune of $2.5 billion in health system funds to address SDOH across multiple sectors (housing, food, education, employment, transportation). Most upstream initiatives were undertaken by large teaching hospitals participating in value-based payment arrangements (96).

Mixed initiatives incorporated both downstream and upstream elements, for example, one food insecurity initiative (62) focused on connecting food-insecure patients to food programs and enrolling them in public benefits such as the Supplemental Nutrition Assistance Program (SNAP). This initiative also used aggregate data on food insecurity to successfully advocate for a simplification of the state’s SNAP application form, which in turn helped to increase statewide enrollment in the program.

Moving on to the second data item in Table 3, the results revealed that a majority (74%) of the initiatives used hot spotting to target HRSNs of defined high-risk populations, while 26% relied on screening and referral to address HRSNs of individual patients. An example of hot spotting would be developing a clinical and socio-behavioral prediction model of 30-day hospital readmissions among people with HIV beyond EHR data (60). An example of screening and referral would be low-intensity resource referral to enhance patients’ knowledge and use of community resources (55). It would be relevant to note that hot spotting has potential to lead to downstream, upstream, or mixed initiatives. For example, “enabling services” is a term that is used to refer to initiatives undertaken by healthcare organizations that have the potential to address a combination of HRSNs like food insecurity and access to care structural barriers (SDOH) and are intended to reduce health disparities (76).

With respect to the third data item in Table 3, Although some initiatives (17%) were focused purely on Quality Improvement (QI) or improving care processes for individuals, more initiatives sought to reduce social risk and undertake Population Health Management (PHM) in their communities (41%) or did both, i.e., combined QI and PHM (41%). An example of a QI initiative would be “fidelity of a screening instrument for HRSNs in the emergency department during COVID-19 (43).” An example of a PHM initiative would be a “family-centered intervention for depressed older men in primary care (44).” An example of mixed QI and PHM initiative would be one that focuses on improving implementation of an initiative designed to promote population health outcomes, such as implementing group prenatal care for minority women living with HIV at an urban university hospital (65).

Moving on to the fourth data item, most (60%) initiatives relied on built internal capacity vs. external or community partnerships (4%) to address HRSNs. The remaining 36% (25) of the initiatives relied on a mix of internal capacity and external resource referrals or community partnerships. Examples of built internal capacity would be investment in hospital-based food pantries to provide healthy foods or investment in community health worker resources to reduce 30-day hospital readmissions (99). An example of reliance on external or community resources would be a Community Rx trial that sought to provide low-intensity resource referrals to influence patients’ knowledge and use of community resources (55). On the other hand, an example of a hospital-community partnership would be hospital partnership with a community farm to offer healthy food options (67).

With respect to the fifth data item, there was limited emphasis (11%) on addressing health disparities either by way of including specific measures of health disparities or by way of devoting discussion points to the topic. For example, a social-return-on-investment analysis of a hospital’s affordable housing program included specific community-level disparity measures related to crime, safety, homelessness, housing stability, and loneliness among seniors (77).

Table 4 presents the results in response to RQ #4 (use of the EHR system to integrate SDOH data into care practices) and RQ #5 (challenges encountered by hospital initiatives to address SDOH). Figure 4 (bottom left) also provides a graphical summary of results for RQ#4 (EHR use for SDOH). The results revealed that only 34% (24) of the initiatives fulfilled Step 1, i.e., used the EHR to collect, store, or present, patient or community level SDOH data. Of the 24 initiatives, only 5 incorporated community-level SDOH data in the EHR. Also, 14 out of the 24 initiatives fulfilled Step 2, integrated SDOH data into care workflows by sharing patient-level SDOH data with the care team and enabling the care team to discuss it with patients. Fewer, 3 out of 24 accomplished Step 3, EHR-based automated support based on SDOH data, while more initiatives, 14 out of 24 accomplished Step 4 of evaluating the impact of integrating SDOH into healthcare delivery.

**Table 4:** EHR use and other challenges reported in addressing SDOH.

Integration of SDOH data into care practices through the EHRs				
**Step to be followed**	**Documented Adherence**	**Frequency**	**Percentage**
Step 1: Collected/organized patient-reported or community-level SDOH data in the EHR (*N* = 70)		Yes	24	34.29%
		Not Documented	46	65.71%
		Total	70	100%
Step 2: Integrated SDOH data into care workflows using the EHR (*N* = 24)	Were patient-level SDOH data shared with the care team?	Yes	19	79.17%
		Not Documented	5	20.83%
		Total	24	100%
	Did the care team discuss SDOH results with patients?	Yes	14	58.33%
		Not Documented	10	41.67%
		Total	24	100%
Step 3: Developed EHR-based automated support and action based on SDOH data (*N* = 24)		Yes	3	12.5%
		Not Documented	21	87.5%
		Total	24	100%
Step 4: Evaluated the impact of integrating SDOH into care delivery (*N* = 24)		Yes	14	58.33%
		Not Documented	10	41.67%
		Total	24	100%
**Other challenges reported in addressing SDOH**				
			**Frequency**	**Percentage**
Implementation challenges			12	17.14%
Organizational challenges			8	11.43%
Limited best-practice and research evidence			3	4.28%
Limited potential for replicability			3	4.28%
Insufficient community-level data			1	1.43%
Not Discussed			43	61.43%
Total			70	100%

Regarding challenges encountered (RQ#5), the articles reported a range of challenges encountered by hospitals addressing SDOH. Figure 3 (right) provides a graphical summary of challenges reported. The most frequently reported were implementation and organizational challenges. Implementation challenges included challenges related to service integration across sectors and, other barriers to resources access such as patient hesitancy to use services due to social stigma. Organizational challenges included limited institutional support for screening and referral, limited provider buy-in for documentation of social needs, insufficient professional training, limited English proficiency of patients leading to incomplete screening; challenges in tracking referrals and inability to verify self-reported data. There were also challenges reported of limited evidence on the systemic impact of SDOH initiatives, e.g., evidence on just one dimension of the Triple Aim, like patient satisfaction, without any data on population health outcomes or cost reduction. Related to this are issues of limited potential for replicability of interventions for addressing SDOH, insufficient community-level data on the magnitude of social need prevalence and the effectiveness of community resource referrals.

## Discussion

### Summary of results

With respect to article characteristics, most articles included in the review (73%) were published during or after 2020. Overall, the results revealed that the peer-reviewed literature on the topic is characterized by research initiatives (79%), including hospital and health system-led clinical trials (40%) or cohort studies (17%) to address downstream health-related social needs (HRSNs) of patients, with an emphasis on improving clinical outcomes (e.g., outcomes of chronic diseases or high-risk healthcare utilization).

The results related to descriptive characteristics of hospital and health system initiatives to address SDOH revealed that more initiatives were undertaken by non-safety net hospitals (51%), including academic health centers and nonprofit hospitals or health systems, compared to safety-net hospitals (30%), including SNHCs, FQHCs, CHCs, and MSHs. Most initiatives addressed two or more (multiple) SDOH concurrently, and food, housing, education, transportation, and financial security, were among the most frequently addressed health-related social needs. The top three Healthy People domains were Social and Community Context, followed by Neighborhood and Built Environment, and Economic Stability. By comparison, fewer initiatives addressed Healthcare Access and Quality, which was one of the IHI-recommended SDOH areas. Other IHI-recommended areas that received little or no attention were climate and decarbonization, voting rights, immigrant needs, and loneliness and social support among the older adults. Chronic diseases and high-risk for healthcare utilization were among the conditions that received the most attention, and many initiatives offered community health worker services, including navigation, coordination, and self-management support services to address social needs and improve clinical outcomes of targeted populations.

By emphasis area, most initiatives 74% were downstream efforts to address HRSNs either through hot spotting or through screening and referral. Only 9% were upstream efforts to address structural SDOH at a community level, including direct investments (in building up community level structural SDOH, e.g., housing) or policy advocacy or community partnerships to promote population health. The remaining 17% were mixed initiatives exhibiting both downstream and upstream elements. Most upstream initiatives were undertaken by large teaching hospitals participating in value-based payment arrangements. Most initiatives (74%) relied on hot spotting (targeting high-risk patients in the hospital or community) compared to screening and referral for social needs (26%), and more initiatives (41%) sought to undertake Population Health Management (PHM) and reduce social risk in their communities compared to relying purely on Quality Improvement (17%). Most initiatives (60%) also relied on built internal capacity compared to community partnerships (4%) to provide services, and few initiatives (115) sought to reduce health disparities or promote health equity.

Findings related to use of the EHR system to integrate SDOH data into care practices revealed considerable shortcomings with only 34% (24) initiatives using the EHR to collect, store, or present, patient or community level SDOH data. Of the 24 initiatives, only 14 integrated SDOH data into care workflows by sharing patient-level SDOH data with the care team and enabling the care team to discuss it with patients. Fewer, 3 out of 24 incorporated EHR-based automated support based on SDOH data.

### Summary assessment of progress and gaps in the adequacy and distribution of initiatives

Results of this scoping review of h peer-reviewed literature on hospital and health systems initiatives to address SDOH, revealed progress on some fronts. Hospital and health systems’ emphasis on hot spotting is a promising trend since it signals efforts on their part to reduce social risk and improve outcomes for high-risk patients. Moreover, the higher emphasis on PHM compared to QI also indicates efforts on the part of hospitals to reach beyond simply improving care processes for individuals, QI, to reducing social risk and improving population health outcomes (PHM) in their communities. Many hospitals and health system initiatives have sought to address health-related social needs of chronic disease patients and patients at high risk for healthcare use, including readmission. Also, most initiatives have sought to address two or more HRSNs at a time (e.g., food and housing insecurity). This is meaningful because social needs such as food, housing, and transportation often do not exist in isolation. They are inextricably linked. The high reliance on internal capacity to conduct SDOH initiatives is commendable since it indicates commitment by hospitals to addressing SDOH, however, it also reveals challenges in building hospital-community partnerships for taking those initiatives to the next level.

By comparison, several gaps were noted on other fronts. Results showed that more initiatives were undertaken by non-safety-net hospitals compared to safety-net hospitals. In other words, a lower proportion of initiatives emanated from hospitals serving minority, vulnerable, and health disparate communities. Also, most initiatives were hospital-led research initiatives, including clinical trials to address SDOH. Only a third of all initiatives used the EHR to collect, store, or present SDOH data. When combined, these findings reveal a missed opportunity for hospitals to address social needs holistically through screening and referral using the EHR system. They also indicate limited potential for transferability of initiatives to safety-net hospitals serving vulnerable populations, where clinical trials may not be as abundant. Regarding types of SDOH addressed, results revealed that there were no hospital initiatives that sought to address some of the IHI-recommended SDOH areas, including climate and decarbonization, voting rights, and immigrant needs, although there were two initiatives that sought to address social support among the older adults. Based on industry recommended SDOH types, results reveal that these are areas that hospitals and health systems could focus on, moving forward.

Another gap noted was in the limited number of upstream initiatives and the types of hospital organizations undertaking these initiatives. Only 9% were upstream initiatives seeking to address structural SDOH at a community level, and these high-impact initiatives were mostly restricted to large teaching hospitals participating in value-based payment arrangements. Other gaps noted were the significantly lower reliance on community partnerships or policy advocacy to promote population health, compared to internal capacity, and the limited emphasis on addressing health disparities. Challenges frequently reported by hospital and health system-led initiatives to address SDOH included implementation challenges, organizational challenges, insufficient community-level data on social need prevalence limited potential for replicability of interventions for addressing SDOH, and limited evidence on the systemic impact, including cost savings from SDOH initiatives.

### Implications for practice, policy, and future research

Most hospital and health system led initiatives to address SDOH were research initiatives (including clinical trials) that relied on technologies independent of the EHR to capture SDOH data. Hospital-initiated clinical trials do serve a purpose in addressing HRSNs of defined populations through targeted interventions. However, the lack of integration of SDOH data into EHR systems and care practices could pose a serious challenge to hospitals’ abilities to holistically address the social needs of their communities, promote population health, and leverage the cost savings from addressing the underlying determinants of health (28, 33, 38).

This type of SDOH-EHR integration, moreover, would be of utmost importance for safety-net facilities where clinical trials may be less of an option. Healthcare payers and policymakers need to recognize this pattern and incentivize hospitals of all types to invest in the use of EHR systems to integrate SDOH data into care practices.

With respect to the emphasis of hospital-led initiatives, upstream initiatives to address SDOH have the potential to address structural determinants of health and reducing health disparities in communities, however, the results showed that upstream initiatives were mostly undertaken by large teaching hospitals that participated in value-based payment arrangements with US federal payers. This suggests that expanding the scope of value-based payment models to include the reduction of health disparities (in addition to improving outcomes) could help to accelerate the pace of hospital efforts to address SDOH (96). Also, given the proclivity of hospitals and health systems to rely on built internal capacity vs. community partnerships, policymakers could play a role in fostering hospital-community (23) partnerships to address SDOH through grants, contracts, and tax-based incentives. Concurrently, policymakers could play a role in incentivizing research seeking to enhance the replicability of SDOH interventions and research seeking to assess the systemic impact (including cost savings) from hospital and health system led interventions to address SDOH.

From a practice perspective, the level of resources that hospitals allocate to addressing SDOH would depend on the prevalence of social need in the community. In addition to investing in identifying this need, any hospital or health system interested in addressing SDOH would see value in investing in EHR systems to integrate SDOH data into care practices. Existing research (28, 32, 33) has demonstrated that investment in SDOH-EHR integration has potential to ensure that the social needs of patients and communities are addressed in a holistic rather than fragmented manner. Moreover, given the significantly lower reliance on community partnerships and the implementation challenges inherent in multisectoral service coordination, results suggest that it may be best for hospitals and health systems to begin small with downstream efforts to address HRSNs and incrementally build up their efforts to address upstream social risk through policy advocacy and community partnerships.

Future research could be aimed at addressing the gaps identified in adequacy and distribution of hospital initiatives to address SDOH, including the use of EHRs to integrate SDOH into care practices and the development of hospital-community partnerships, especially among safety-net hospitals. Future research could also be targeted toward addressing the gaps in IHI-recommended SDOH areas such as climate and decarbonization, voting rights, and in addressing challenges encountered by hospital-led initiatives, including organizational and implementation challenges, insufficient data on social need prevalence and the effectiveness of community resource referrals, and limited evidence on the systemic impact and cost savings of SDOH interventions.

### Strengths and limitations

A key strength of this paper lies in addressing a gap in the literature related to characteristics of hospital and health system initiatives to address SDOH in the US to understand the progress and gaps in this area. The scoping review was guided by evidence-based criteria for scoping reviews developed by the JBI and the internationally accepted PRISMA guidelines for scoping reviews. A clear rationale for use of scoping review (vs. other types of review techniques) is provided at the outset, and the review questions are aligned with the broader research objective. Consistent with the rationale, the review entailed use of an integrated framework of Healthy People SDOH domains and industry recommended SDOH types for hospitals to inform a comprehensive search of the peer-reviewed literature for eligible individual sources of evidence.

One limitation was that other avenues for literature searches were not leveraged to identify additional articles, e.g., “gray literature searches” of industry publications, online resources, unpublished manuscripts, and conference papers. Also, to ensure manageable search results, the historical search timeline could not be extended to 2010 to capture progress made on this topic since the introduction of the US Affordable Care Act. Additionally, as indicated under study purpose, the focus of this review is on understanding “what” hospitals are doing to address SDOH, which in turn helps to understand the progress and gaps related to the adequacy and distribution of hospital initiatives to address SDOH in the US. On the other hand, the question of “how effectively” hospitals are addressing SDOH is beyond the scope of this study. As such, this review provides limited insights into the effectiveness (or impact on outcomes) of hospital and health system-led interventions to address SDOH. Nevertheless, it would be relevant to note that the question of effectiveness is broadly addressed in the context of EHR use to integrate SDOH data into care practices, by applying an established four-step framework for assessing the effectiveness of hospital SDOH initiatives in this area. As a next step, the authors plan to leverage the foundation provided by this paper on key characteristics of hospital SDOH initiatives, to conduct an independent systematic review devoted to addressing the question of the effectiveness of hospital initiatives to address SDOH for future publication.

## Conclusion

This paper presents the results of a scoping review of 70 peer-reviewed articles to describe the key characteristics of hospital and health system led initiatives to address SDOH. Results revealed that the peer-reviewed literature on this topic is largely characterized by hospital and health system-led clinical trials to address downstream health-related social needs (HRSNs) with an emphasis on improving clinical outcomes. Hospital and health systems’ focus on hot spotting or targeting social needs of high-risk patients is a promising trend since it signals efforts on the part of hospitals and health systems to reduce social risk and improve outcomes for defined populations. Also, most hospital and health system initiatives have addressed two or more social needs at a time, which is commendable, because social needs are often inextricably linked (e.g., housing and food insecurity) and need to be addressed concurrently for meaningful progress. The influence of the evolving policy environment is evident in the results indicating an emphasis by hospitals on addressing the health-related social needs of patients with (1) chronic disease conditions and (2) high-risk for healthcare utilization. Since the introduction of the ACA, both these areas have been targeted for value-based payment reform by the CMS and private payers. Also, the fact that most articles were published during the pandemic and post-pandemic period may be viewed as a reflection of the evolving policy environment, including the growing attention to the reduction of health disparities and promotion of health equity at a national level.

With respect to gaps, results revealed that a larger proportion of the initiatives were undertaken by non-safety-net hospitals, compared to by safety-net hospitals. Most initiatives were designed as clinical trials to address SDOH, however, most trials used EHR-independent technologies to collect and report SDOH data. The high reliance on built internal capacity, while commendable, also reveals challenges in developing community partnerships to address SDOH. Policymakers must create incentives for hospitals to invest in (1) integrating SDOH data into EHR systems and (2) harnessing community partnerships to promote population health. Also, since most upstream initiatives were undertaken by large teaching hospitals that participated in value-based payment arrangements, enhancing the scope of value-based payment models to include the reduction of health disparities could help to accelerate the pace of hospital efforts to address SDOH. Future research could be aimed at addressing the gaps in hospital and health system initiatives to address SDOH, including the use of EHRs to integrate SDOH into care practices, the development of hospital-community partnerships to address health-related social needs at the community level, the types of SDOH addressed based on IHI recommendations, and the generation of evidence related to the systemic impact and cost savings of hospital and health system-led SDOH interventions.

## Data availability statement

The original contributions presented in the study are included in the article/Supplementary material, further inquiries can be directed to the corresponding author.

## Author contributions

PR: Conceptualization, Data curation, Formal analysis, Investigation, Methodology, Project administration, Resources, Software, Supervision, Validation, Visualization, Writing – original draft, Writing – review & editing, Funding acquisition. AT: Data curation, Formal analysis, Methodology, Validation, Writing – review & editing. DS: Data curation, Formal analysis, Methodology, Supervision, Validation, Writing – review & editing. KK: Data curation, Formal analysis, Methodology, Validation, Writing – review & editing. KR: Data curation, Formal analysis, Methodology, Writing – review & editing. HJ: Data curation, Formal analysis, Methodology, Writing – review & editing. LG: Funding acquisition, Validation, Writing – review & editing.
