# Supplementary material for: Characteristics of hospital and health system initiatives to address social determinants of health in the United States: a scoping review of the peer-reviewed literature
Source: Front Public Health. 2024 May 30;12:1413205. doi: 10.3389/fpubh.2024.1413205 (PMC11173975; doi:10.3389/fpubh.2024.1413205)
Supplement: Supplementary file 1 [file Data_Sheet_1.docx]

**Review Protocol**

**Characteristics of Hospital & Health System Initiatives to Address Social Determinants of Health (SDOH) in the United States: A Scoping Review of the Peer-Reviewed Literature**

**Background**

Despite the incentives and provisions created for hospitals by the US Affordable Care Act related to value-based payment and community health needs assessments, concerns remain regarding the adequacy and distribution of hospital efforts to address SDOH. This scoping review of the peer-reviewed literature identifies the key characteristics of hospital/health system initiatives to address SDOH in the US, to gain insight into the progress and gaps.

***Review questions.***

The objective of this review is to describe the characteristics of hospital and health system initiatives to address SDOH. The specific review questions are outlined below.

1. What are the article characteristics of hospital and health system initiatives to address SDOH, by publication year, article type, methodology, and inclusion of outcome measures?
2. What are the descriptive characteristics of hospital and health system initiatives to address SDOH, e.g., by the number and type of SDOH addressed, type of hospital organization, US geographic region, conditions targeted, and services provided?
3. What is the emphasis of hospital/health system initiatives to address SDOH with respect to the following characteristics?
4. *Addressing downstream HRSNs vs upstream SDOH.*
5. *Screening & referral vs. hot spotting.*
6. *Quality improvement vs. population health management.*
7. *Reliance on internal capacity vs. community partnerships to provide services.*
8. *Reducing health disparities and/or promoting health equity.*
9. Have hospitals and health systems utilized the Electronic Health Record (EHR) to integrate SDOH data into care practices?
10. What major challenges have been reported in hospital and health system initiatives to address SDOH?

**Scoping review rationale**

According to Sucharew and Macaluso (2019), scoping reviews can be useful for answering broad questions, such as “What information has been presented on this topic in the literature?” which in turn is fully consistent with what this review seeks to accomplish [25]. To-date, there have been no comprehensive reviews of peer-reviewed literature to obtain the *scope of knowledge on characteristics of hospital and health system-led initiatives to address SDOH in the US*. This paper seeks to address this gap, and the review questions in turn, are aligned with this purpose.

**Scoping review methodology**

Framework for the scoping review (PRISMA-ScR)

PRISMA-ScR criteria were used to inform a scoping review of the literature [26]. Three academic databases were searched for eligible sources of evidence from 01.01.2018 through 06.30.2023. The review protocol (**Appendix 1**) was developed based on guidelines for scoping reviews provided by the Joanna Briggs Institute (JBI) [27]. The protocol was not registered. The PRISMA-ScR checklist is included in **Appendix 2**.

Information Sources

This scoping review sought to identify published research articles, review articles, and case reports to address the review questions. Three academic databases, PubMed, ABI/Inform (Pro Quest), and Academic Search Premier were searched for eligible articles over a 5+ year timeframe between 01.01.2018 and 06.30.2023. Since COVID-19 is known to have played a significant role in elevating the topic of SDOH to center stage in health policy debates [2,10], papers published over the period spanning two years pre/post the pandemic year (2020) were considered for inclusion. The article search was conducted in September 2023. Given the topic of interest, the three forementioned academic databases were selected to ensure maximum coverage across biomedical science, clinical practice, social science, and health policy domains.

Search Strategy

As mentioned earlier, this paper integrates the Healthy People 2030 SDOH framework and the IHI SDOH types to inform the search strategy **(Figure 1**). To identify individual SDOH search terms under the Healthy People SDOH domains, this study leveraged standardized SDOH terminology and keywords representing specific social and behavioral determinants of health in Healthy People, as well as published search algorithms from existing reviews that utilized the Healthy People framework [18,28]. These terms were then supplemented with individual search terms to capture the IHI SDOH types [21].

Next, Boolean search operators were used to combine all individual SDOH terms with a set of search terms to capture the care setting of interest. As mentioned earlier, 57% of community hospitals in the US belong to health systems. In addition, over 220 nonprofit community hospitals are classified as academic medical centers or academic health centers [5]. Moreover, since the distribution of SDOH initiatives by type of hospital organization is of interest (e.g., nonprofit hospitals vs safety-net health centers), it would also be important to capture initiatives being undertaken by safety net health centers. Therefore, the search terms of “hospital,” “health system,” “health center,” and “medical center,” to capture the care setting of interest were all relevant to this study. Finally, the forementioned two sets of search terms were combined with MeSH terms (Medical Subject Headings) to capture the broader concept of social determinants of health. The full electronic search strategy used on PubMed is included as an example, in **Appendix 3**.

Eligibility Criteria

This scoping review considered research articles, review articles, and case reports that were published in peer-reviewed journals in English, were based in the United States. As mentioned earlier, papers published over a 5+year period between 01.01.2018 and 06.30.2023 were considered for inclusion. Research articles considered for inclusion were based on empirical data collected from clinical trials, cohort studies, case-control studies, secondary data analysis, cross-sectional studies, and other types of quantitative, qualitative, and mixed-method studies. Also considered for inclusion were case reports, systematic reviews, and scoping reviews.

This scoping review excluded: **1)** articles published outside the selected date range. It also excluded **2)** articles that were not published in peer-reviewed journals (e.g., conference papers, working papers, wire feeds, reports, abstracts, books, trade journals, dissertations, theses, and magazines and other non-scholarly sources). Also excluded were **3)** articles that were not published in English language. Additionally, this review excluded **4)** articles that were not based in the US. In other words, one or more of the authors’ affiliations needed to be based in the US, and the article needed to describe a hospital or health system-led initiative to address SDOH that was based in the US. This review also excluded **5)** articles that were not of an eligible article type, such as study protocols, editorial articles, and discussion papers. Additionally, **6)** papers that were not available in full text either through an open source or the original publishing source were excluded from consideration. Also excluded from this review were **7)** articles that were determined to be outside the scope of the study. The criteria used for determination of review scope are described below. Lastly, this review excluded **8)** articles that did not meet critical appraisal criteria outlined by the JBI [27] and the Mixed-Method Appraisal Tool (MMAT) [29].

To elaborate on study scope (Category #7 above), to be considered within the study scope, the article needed to describe a hospital or health system-led initiative based in the US to address SDOH at the patient or community level. By comparison, articles determined to be outside the scope of the study included research initiatives undertaken by hospitals or health systems, based on literature reviews or secondary analysis of national, statewide, or regional databases, to describe broad socioeconomic challenges and put forth strategies, policies, and programs for addressing them. Examples include a systematic review of technologies for prehospital communication and coordination [30]; analysis of the national cardiovascular data registry to examine neighborhood socioeconomic disadvantage and care after myocardial infarction [31]; and cross-sectional validation of the PROMIS-Preference scoring system by its association with SDOH [32]. It would be relevant to note that although review articles were considered for inclusion, most reviews were deemed to be outside the scope, since they pertained to the broader impact of SDOH on specific conditions (e.g., diabetes) or outcomes (readmissions), often assessed at a national level. Like reviews, most analytic studies were multi-year, state, or national-level analyses of SDOH that were determined to be outside the study scope. By comparison, most clinical trials were included since they pertained to hospital or health system-led initiatives to address SDOH.

Process for selecting sources of evidence.

The three academic databases used for the article search were equipped with search capabilities that enabled article exclusion based on many of the review’s exclusion criteria, prior to downloading articles for duplicate removal and title & abstract screening. This included the exclusion of articles published outside of the selected date range for this study; articles that were not published in peer-reviewed journals; articles that were not in English language; articles that were not based in the US; articles that were not of an eligible article type; and articles that were not available in full text either from an open source or the original publishing source.

All articles identified following the database search, were collated, and uploaded into a reference management system (Zotero 5.0) for the removal of duplicates. After duplicate removal, article titles and abstracts were screened for potential inclusion, based on the eligibility criteria. Lastly, articles identified for inclusion were retrieved in full text for further review. Articles selected for inclusion following full-text review were subjected to critical appraisal using JBI checklists (for clinical trials, cross-sectional studies, qualitative studies, and review articles) [27]. The MMAT was used to appraise mixed-method studies [29]. Only articles that met the critical appraisal criteria were identified for final inclusion in the scoping review.

Process for charting data items.

All included articles were reviewed to retrieve and chart four categories of data (Data Categories 1-4) to address the research questions, as shown in **Appendix 4, Charts 1-4 respectively**.

**Data Category #1**: data items for characterizing article characteristics on hospital and health system initiatives to address SDOH, aligned with Research Question (RQ) #1. This included the publication year, article type, methodology, and inclusion of outcome measures.

**Data Category #2**: data items for capturing the descriptive characteristics of hospital initiatives to address SDOH, aligned with RQ #2, including the number and type of SDOH addressed, type of hospital organization, US geographic region, targeted diseases/conditions, and services offered as part of the initiative.

**Data Category #3:** data items for capturing additional characteristics of hospital initiatives to address SDOH, aligned with RQ #3, i.e., emphasis on: 1) upstream vs. downstream initiatives; 2) screening & referral vs. hot spotting, 3) quality improvement vs. population health management, 4) reliance on internal capacity vs. community partnerships; and 5) reducing health disparities and/or promoting health equity. First, as discussed earlier, hospital and health systems initiatives could take the form of downstream initiatives to address HRSN at the individual level or upstream initiatives to address structural SDOH at the community level. Second, hospital and health system initiatives could take the form of 1) screening & referral to address unmet social needs; or 2) a hot spotting approach to target a defined population at-risk, be it patients who visit the facility, e.g., chronic disease patients, or high utilizers within the community.

Third, hospital and health system initiatives could be designed as Quality Improvement (QI) initiative or Population Health Management (PHM) initiative or a mix of both. The focus of QI is on enhancing processes and systems within the organization to deliver higher quality care to individual patients, while PHM focuses on improving the health outcomes of a defined population by reducing social risk. PHM involves analyzing data to understand health outcomes, trends, and disparities within the population, implementing interventions, and measuring impact.

Fourth, hospitals could rely on built internal capacity (e.g., hospital-based food pantries) or rely on community partnerships to provide services. (e.g., partnership with a community farm to offer healthy foods). The fifth item captures any special emphasis on the part of hospital and health system SDOH initiatives to reduce health disparities or improving health equity.

**Data Category #4** This includes data items to capture hospital adherence to an existing four-step framework for using the EHR to integrate SDOH into care practices, in alignment with RQ#4 [22,23]. **Appendix 4, Chart 3** summarizes adherence to each of the four steps with a yes/no measure, while **Appendix 5** (raw dataset) includes a more detailed assessment of adherence to various sub steps within each of these four steps [23]. Also included in this category are challenges encountered by hospitals in addressing SDOH, in alignment with RQ#5. The data items described under all four data categories were charted in multiple spreadsheet templates included in **Appendix 5**, the raw dataset for the study.

Process for synthesizing results.

The data collected and charted from articles were summarized using counts, aggregates, and proportions for analysis and interpretation based on the review questions. This process helped to synthesize the evidence related to the progress and gaps in hospital initiatives to address SDOH as well as the state of the science pertaining to hospital initiatives to address SDOH.
