# Supplementary material for: Characteristics of hospital and health system initiatives to address social determinants of health in the United States: a scoping review of the peer-reviewed literature
Source: Front Public Health. 2024 May 30;12:1413205. doi: 10.3389/fpubh.2024.1413205 (PMC11173975; doi:10.3389/fpubh.2024.1413205)
Supplement: Supplementary file 3 [file Data_Sheet_3.docx]

**Appendix 3**

| **Search Terms** | **Results** |
| --- | --- |
| (((((((((((((hous*[Title/Abstract]) OR (unemploy*[Title/Abstract])) OR (underemploy*[Title/Abstract])) OR (poverty[Title/Abstract])) OR (low income[Title/Abstract])) OR (public housing[Title/Abstract])) OR (salary[Title/Abstract])) OR (wage*[Title/Abstract])) OR (home ownership[Title/Abstract])) OR (internet access[Title/Abstract])) OR (cell* phone access[Title/Abstract])) OR (technology[Title/Abstract])) OR (((((((((((((((((((((((social[Title/Abstract]) AND ((((isolat*[Title/Abstract]) OR (integrat*[Title/Abstract])) OR (support*[Title/Abstract])) OR (cohes*[Title/Abstract])) OR (engage*[Title/Abstract]))) OR (support network*[Title/Abstract])) OR (support system*[Title/Abstract])) OR (discriminat*[Title/Abstract]))) OR (racism[Title/Abstract])) OR (sexism[Title/Abstract])) OR (race/ethnicity[Title/Abstract])) OR (sex/gender[Title/Abstract])) OR (prison[Title/Abstract])) OR (jail[Title/Abstract])) OR (incarceration[Title/Abstract])) OR (“voting right” [Title/Abstract])) OR (stigma[Title/Abstract])) OR (stress*[Title/Abstract])) OR (homophob*[Title/Abstract])) OR (LGBT*[Title/Abstract])) OR (accultur*[Title/Abstract])) OR (foreign born[Title/Abstract])) OR (trauma[Title/Abstract])) OR (adverse child* experience*[Title/Abstract])) OR ((((((((((((((((((((((((transportation[Title/Abstract]) OR (safe*[Title/Abstract])) OR (crime[Title/Abstract])) OR (violen*[Title/Abstract])) OR (food access[Title/Abstract])) OR (neighborhood[Title/Abstract])) OR (built environment[Title/Abstract])) OR (climate[Title/Abstract])) OR (decarbonization[Title/Abstract])) OR (decarbon*[Title/Abstract])) OR (carbon[Title/Abstract)) OR (food inqual*[Title/Abstract])) OR (food insecur*[Title/Abstract])) OR (access to food*[Title/Abstract])) OR (food desert[Title/Abstract])) OR (geograph*[Title/Abstract])) OR (park*[Title/Abstract])) OR (urban[Title/Abstract])) OR (rural[Title/Abstract])) OR (domestic violence[Title/Abstract])) OR ("intimate partner violence"[Title/Abstract])) OR (residen* characteristics[Title/Abstract])) OR (resident* segregation[Title/Abstract]))) OR (((((((((((((child* develop*[Title/Abstract]) OR (literacy[Title/Abstract)) OR (health literacy[Title/Abstract])) OR (health numeracy[Title/Abstract])) OR (GED[Title/Abstract])) OR ("general education development"[Title/Abstract])) OR (school read*[Title/Abstract])) OR (child* education[Title/Abstract])) OR (high school graduat*[Title/Abstract])) OR (language*[Title/Abstract])) OR (early child* education[Title/Abstract])) OR ("English as a second language"[Title/Abstract])) OR (ESL[Title/Abstract])) OR (((((health* coverage[Title/Abstract]) OR (insurance[Title/Abstract])) OR (insur*[Title/Abstract])) OR ("access to care"[Title/Abstract])) OR (Medicaid[Title/Abstract])) OR (uninsur*[Title/Abstract]))  AND  ((((hospital[Title/Abstract]) OR (health system[Title/Abstract])) OR (medical center[Title/Abstract])) OR (health center[Title/Abstract]))  AND  (((social[Title/Abstract]) AND ((((((((((((determinant*[Title/Abstract]) OR (determinants of health[Title/Abstract])) OR (risk*[Title/Abstract])) OR (need*[Title/Abstract])) OR (disadvantage*[Title/Abstract])) OR (depriv*[Title/Abstract])) OR (disparit*[Title/Abstract])) OR (inequit*[Title/Abstract])) OR (gradient*[Title/Abstract])) OR (inequalit*[Title/Abstract])) OR (capital[Title/Abstract])))) OR (social determinants of health[MeSH Terms]))  AND  (("2018/01/01"[Date - Publication] : "2023/06/30"[Date - Publication])) | [8,772](https://pubmed-ncbi-nlm-nih-gov.unh-proxy01.newhaven.edu/?term=longquerycdd703b2125f89e0fd7a&ac=no&sort=relevance) |
| Date and Language Filter | [8,505](https://pubmed-ncbi-nlm-nih-gov.unh-proxy01.newhaven.edu/?term=longquery9c4317688bf76ddb0cd7&filter=lang.english&ac=no) |
| Date, Language, and Full Text Filter | [8,342](https://pubmed-ncbi-nlm-nih-gov.unh-proxy01.newhaven.edu/?term=longquery9c4317688bf76ddb0cd7&filter=simsearch3.fft&filter=lang.english&ac=no) |
| Date, Language, Full Text, and Article Type Filter | [**1,564**](https://pubmed-ncbi-nlm-nih-gov.unh-proxy01.newhaven.edu/?term=longquery9c4317688bf76ddb0cd7&filter=simsearch3.fft&filter=pubt.casereports&filter=pubt.classicalarticle&filter=pubt.clinicalstudy&filter=pubt.clinicaltrial&filter=pubt.comparativestudy&filter=pubt.controlledclinicaltrial&filter=pubt.evaluationstudy&filter=pubt.introductoryjournalarticle&filter=pubt.meta-analysis&filter=pubt.multicenterstudy&filter=pubt.observationalstudy&filter=pubt.review&filter=pubt.systematicreview&filter=lang.english&ac=no) |
| **Total Downloaded to Zotero** | **1,564** |

**Search Strategy on** [**PubMed**](https://pubmed.ncbi.nlm.nih.gov/)
