# Supplementary material for: Characteristics of hospital and health system initiatives to address social determinants of health in the United States: a scoping review of the peer-reviewed literature
Source: Front Public Health. 2024 May 30;12:1413205. doi: 10.3389/fpubh.2024.1413205 (PMC11173975; doi:10.3389/fpubh.2024.1413205)
Supplement: Supplementary file 4 [file Data_Sheet_4.docx]

**Appendix 4: Data Charting on Individual Sources of Evidence in Response to Review Questions**

**70 Individual Article Reference Numbers in Manuscript [23, 24, 27, 34, 43-108]**

**Table 1: Data Charting in Response to RQ #1**

| **Record #** | **Article Name [REF#]** | **Publication Year** | **Article Type** | **Methodology** | **Does the study include any type of outcome measures? (Y/N)** | **Does the study include clinical outcome measures? (Y/N)** | **Does the study include social outcome measures? (Y/N)** |
| --- | --- | --- | --- | --- | --- | --- | --- |
| **1** | Missed psychosocial risk factors during routine preoperative evaluations are associated with increased complications after elective cancer surgery [45]. | 2019 | Case Study/Report | Case Report | Yes | Yes | Yes |
| **2** | Outpatient Palliative Care for Noncancer Illnesses: One Program's Experience with Implementation, Impact, and Lessons Learned [46]. | 2022 | Case Study/Report | Case Report | Yes | Yes | No |
| **3** | Financial Incentives for Smoking Cessation in Hospitalized Patients: A Randomized Clinical Trial [34]. | 2020 | Research | Randomized Controlled Trial | Yes | Yes | No |
| **4** | Real-World Disparities in Remote Follow-Up of Cardiac Implantable Electronic Devices and Impact of the COVID-19 Pandemic: A Single-Center Experience [47]. | 2023 | Case Study/Report | Case Report | Yes | Yes | Yes |
| **5** | Implementing an EHR-based Screening and Referral System to Address Social Determinants of Health in Primary Care [48]. | 2019 | Research | Observational Study | Yes | No | Yes |
| **6** | Documentation and review of social determinants of health data in the EHR: measures and associated insights [49]. | 2021 | Case Study/Report | Case Report | No | No | No |
| **7** | Effect of Community Health Workers on 30-Day Hospital Readmissions in an Accountable Care Organization Population: A Randomized Clinical Trial [50]. | 2021 | Research | Randomized Controlled Trial | Yes | Yes | No |
| **8** | The impact of enhancing self-management support for diabetes in Community Health Centers through patient engagement and relationship building: a primary care pragmatic cluster-randomized trial [51]. | 2022 | Research | Randomized Controlled Trial | Yes | Yes | Yes |
| **9** | Online Telehealth Delivery of Group Mental Health Treatment Is Safe, Feasible, and Increases Enrollment and Attendance in Post-9/11 U.S. Veterans [52]. | 2022 | Research | Randomized Controlled Trial | Yes | Yes | No |
| **10** | Computerized Intervention in Primary Care for Women Veterans with Sexual Assault Histories and Psychosocial Health Risks: A Randomized Clinical Trial [53]. | 2022 | Research | Randomized Controlled Trial | Yes | Yes | No |
| **11** | Bridge to Health/Puente a la Salud: a pilot randomized trial to address diabetes self-management and social needs among high-risk patients [54]. | 2022 | Research | Randomized Controlled Trial - | Yes | Yes | No |
| **12** | Impact of a Low-Intensity Resource Referral Intervention on Patients' Knowledge, Beliefs, and Use of Community Resources: Results from the Community Rx Trial [55]. | 2020 | Research | Randomized Controlled Trial | Yes | Yes | No |
| **13** | Patient navigation to address sociolegal barriers for patients with cancer: A comparative-effectiveness study [56]. | 2022 | Research | Randomized Controlled Trial | Yes | Yes | Yes |
| **14** | Nurse-led hospital-to-community care, clinical outcomes for people living with HIV and health-related social needs [57]. | 2022 | Research | Retrospective Cohort Study | Yes | Yes | No |
| **15** | Improving Diabetes Care Through Population Health Innovations and Payments: Lessons from Western Maryland [58]. | 2023 | Research | Observational Cohort Study | Yes | Yes | No |
| **16** | Financing Diabetes Care in the U.S. Health System: Payment Innovations for Addressing the Medical and Social Determinants of Health [59]. | 2019 | Review | Review | No | No | No |
| **17** | Clinical and Socio-behavioral Prediction Model of 30-Day Hospital Readmissions Among People with HIV and Substance Use Disorder: Beyond Electronic Health Record Data [60]. | 2019 | Research | Randomized Controlled Trial | Yes | Yes | Yes |
| **18** | Effects of a standardized community health worker intervention on hospitalization among disadvantaged patients with multiple chronic conditions: A pooled analysis of three clinical trials [61]. | 2020 | Research | Randomized Controlled Trial | Yes | Yes | No |
| **19** | Screening for health-related social needs in the emergency department: Adaptability and fidelity during the COVID-19 pandemic [43]. | 2022 | Research | Observational Cohort Study | Yes | Yes | Yes |
| **20** | Food Insecurity in Older Adults in an Integrated Health Care System [62]. | 2018 | Research | Retrospective Cohort Study | Yes | No | Yes |
| **21** | Development and Implementation of a Maryland State Program Providing Hospital Payment Incentives for Reduction in Readmission Disparities [63]. | 2023 | Research | Observational Study | Yes | Yes | Yes |
| **22** | Implementation fidelity to a behavioral diabetes prevention intervention in two New York City safety net primary care practices [64]. | 2023 | Research | Randomized Controlled Trial | Yes | Yes | No |
| **23** | Implementing Centering Pregnancy Group Prenatal Care for Minority Women Living with HIV at an Urban University Hospital [65]. | 2019 | Research | Retrospective Cohort Study | Yes | Yes | No |
| **24** | Management of Isolated Skull Fractures in Pediatric Patients: A Systematic Review [66]. | 2019 | Review | Review | Yes | Yes | Yes |
| **25** | Health Center-Based Community-Supported Agriculture: An RCT [67]. | 2019 | Research | Randomized Controlled Trial | Yes | Yes | Yes |
| **26** | Effects of In-Person Assistance vs Personalized Written Resources About Social Services on Household Social Risks and Child and Caregiver Health: A Randomized Clinical Trial [68]. | 2020 | Research | Randomized Controlled Trial | Yes | Yes | Yes |
| **27** | Feasibility of a family-centered intervention for depressed older men in primary care [44]. | 2019 | Research | Randomized Controlled Trial | Yes | Yes | No |
| **28** | Intensive care management for high-risk veterans in a patient-centered medical home - do some veterans benefit more than others? [69]. | 2023 | Research | Randomized Controlled Trial | Yes | Yes | No |
| **29** | Pragmatic Clinical Trial to Improve Patient Experience Among Adults During Transitions from Hospital to Home: The PArTNER study [70]. | 2022 | Research | Randomized Controlled Trial | Yes | Yes | Yes |
| **30** | Medical-Legal Partnership Effects on Mental Health, Health Care Use, and Quality of Life in Primary Care: A Randomized Clinical Trial [71]. | 2023 | Research | Randomized Controlled Trial | Yes | Yes | No |
| **31** | Predictive Model-Driven Hot spotting to Decrease Emergency Department Visits: A Randomized Controlled Trial [72]. | 2021 | Research | Randomized Controlled Trial | Yes | Yes | No |
| **32** | A care coordinator screening strategy to address health harming legal needs [73]. | 2022 | Research | Qualitative Study | Yes | No | Yes |
| **33** | Evidence that collaborative action between local health departments and nonprofit hospitals helps foster healthy behaviors in communities: a multilevel study [74]. | 2021 | Research | Analytical Cross-sectional Study | Yes | No | Yes |
| **34** | Hospital Partnerships for Population Health: A Systematic Review of the Literature [23]. | 2021 | Review | Systematic Review | Yes | No | Yes |
| **35** | Medicaid Investments to Address Social Needs in Oregon and California [75]. | 2019 | Research | Qualitative Study | Yes | No | Yes |
| **36** | Enabling Services Improve Access to Care, Preventive Services, And Satisfaction Among Health Center Patients [76]. | 2019 | Research | Analytical Cross-sectional Study | Yes | Yes | No |
| **37** | A Social-Return-On-Investment Analysis of Bon Secours Hospital's 'Housing for Health' Affordable Housing Program [77]. | 2021 | Research | Analytical Cross-sectional Study | Yes | No | Yes |
| **38** | Embedding Social Workers in Veterans Health Administration Primary Care Teams Reduces Emergency Department Visits [78]. | 2020 | Research | Quasi-experimental Study | Yes | Yes | No |
| **39** | Project Nurture Integrates Care and Services to Improve Outcomes for Opioid-Dependent Mothers and Their Children [79]. | 2020 | Research | Quasi-experimental Study | Yes | Yes | Yes |
| **40** | Addressing the Poverty Barrier in Collaborative Care for Adults Experiencing Homelessness: A Case-Based Report [80]. | 2020 | Case Study/Report | Case Study | Yes | Yes | Yes |
| **41** | Interprofessional, multitiered daily rounding management in a high-acuity hospital [81]. | 2020 | Case Study/Report | Case Study | Yes | Yes | No |
| **42** | Indianapolis Provider's Use of Wraparound Services Associated with Reduced Hospitalizations and Emergency Department Visits [82]. | 2018 | Research | Analytical Cross-sectional Study | Yes | Yes | Yes |
| **43** | Reasons for Social Work Referrals in an Urban Safety-Net Population: A Natural Language Processing and Market Basket Analysis Approach [83]. | 2021 | Research | Analytical Cross-sectional Study | No | No | No |
| **44** | The Impact of Food Insecurity Screenings and Community Food Resource Referrals for Patients with Type 2 Diabetes [84]. | 2020 | Research | Prospective Cohort Study | Yes | Yes | Yes |
| **45** | The implementation cost of a safety‐net hospital program addressing social needs in Atlanta [85]. | 2021 | Case Study/Report | Case Study | Yes | Yes | Yes |
| **46** | Development of a homelessness risk screening tool for emergency department patients [86]. | 2022 | Research | Prospective Cohort Study | No | No | No |
| **47** | An embedded multiple case study: using CFIR to map clinical food security screening constructs for the development of primary care practice guidelines [87]. | 2022 | Case Study/Report | Case Study | Yes | No | Yes |
| **48** | Conceptualizing the effective mechanisms of a social needs case management program shown to reduce hospital use: a qualitative study [88]. | 2022 | Case Study/Report | Case Study | Yes | Yes | No |
| **49** | Social Needs Screening and Referral Program at a Large US Public Hospital System, 2017 [89]. | 2020 | Research | Qualitative Study | No | No | No |
| **50** | A Nurse-Led Intervention to Address Food Insecurity in Chicago [90]. | 2018 | Case Study/Report | Case Study | Yes | No | Yes |
| **51** | Training Student Volunteers as "Community Resource Navigators" to Integrate Health and Social Care in Primary Care [91]. | 2022 | Research | Qualitative Study | Yes | No | Yes |
| **52** | An In-Clinic Food Pharmacy Addresses Very Low Food Security [92]. | 2020 | Case Study/Report | Case Study | No | No | No |
| **53** | Evidence based processes to prevent readmissions: more is better, a ten-site observational study [93]. | 2021 | Research | Observational Study | Yes | Yes | No |
| **54** | Unmet Social Needs and No-Show Visits in Primary Care in a US Northeastern Urban Health System, 2018–2019 [94]. | 2020 | Research | Analytical Cross-sectional Study | Yes | Yes | No |
| **55** | Cooling The Hot Spots Where Child Hospitalization Rates Are High: A Neighborhood Approach to Population Health [95]. | 2019 | Research | Analytical Time-series Study | Yes | Yes | No |
| **56** | Quantifying Health Systems' Investment in Social Determinants of Health, By Sector, 2017–19 [96]. | 2020 | Review | Scoping Review | Yes | No | Yes |
| **57** | Prescribing Housing: A Scoping Review of Health System Efforts to Address Housing as a Social Determinant of Health [34]. | 2021 | Review | Scoping Review | Yes | No | Yes |
| **58** | The Baltimore Community-Based Organizations Neighborhood Network: Enhancing Capacity Together (CONNECT) Cluster RCT [97]. | 2019 | Research | Randomized Controlled Trial | Yes | Yes | Yes |
| **59** | Effect of Community Health Worker Support on Clinical Outcomes of Low-Income Patients Across Primary Care Facilities: A Randomized Clinical Trial [98]. | 2018 | Research | Randomized Controlled Trial | Yes | Yes | Yes |
| **60** | Food for Thought: A Randomized Trial of Food Insecurity Screening in the Emergency Department [99]. | 2019 | Research | Randomized Controlled Trial | Yes | No | Yes |
| **61** | Effect of a Peer-Led Behavioral Intervention for Emergency Department Patients at High Risk of Fatal Opioid Overdose: A Randomized Clinical Trial [100]. | 2022 | Research | Randomized Controlled Trial | Yes | Yes | No |
| **62** | Patient plus partner trial: A randomized controlled trial of 2 interventions to improve outcomes after an initial implantable cardioverter-defibrillator [101]. | 2019 | Research | Randomized Controlled Trial | Yes | No | Yes |
| **63** | Illustrating the value of social work: results of an open pilot trial of the psychosocial acuity scale in a large urban pediatric hospital [102]. | 2021 | Research | Randomized Controlled Trial | Yes | Yes | No |
| **64** | Using Publicly Available Data to Understand the Opioid Overdose Epidemic: Geospatial Distribution of Discarded Needles in Boston, Massachusetts [103]. | 2018 | Research | Analytical Cross-sectional Study | Yes | Yes | No |
| **65** | Predictors for Poor Linkage to Care Among Hospitalized Persons Living with HIV and Co-Occurring Substance Use Disorder [104]. | 2020 | Research | Randomized Controlled Trial | Yes | Yes | No |
| **66** | Enhanced care planning and clinical-community linkages versus usual care to address basic needs of patients with multiple chronic conditions: a clinician-level randomized controlled trial [105]. | 2020 | Research | Randomized Controlled Trial | Yes | Yes | Yes |
| **67** | Social Risks Among Primary Care Patients in a Large Urban Health System [27]. | 2020 | Research | Analytical Cross-sectional Study | No | No | No |
| **68** | Relationship of Neighborhood Deprivation and Outcomes of a Comprehensive ST-Segment-Elevation Myocardial Infarction Protocol [106]. | 2021 | Research | Observational Cohort Study | Yes | Yes | No |
| **69** | Enhancing Reach Out and Read with a Video and Text Messages: A Randomized Trial in a Low-Income Predominantly Latino Sample [107]. | 2021 | Research | Randomized Controlled Trial | Yes | No | Yes |
| **70** | Social Needs Screening in Hospitalized Pediatric Patients: A Randomized Controlled Trial [108]. | 2023 | Research | Randomized Controlled Trial | Yes | No | Yes |

**Table 2: Data Charting in Response to RQ#2 Descriptive Characteristics**

| **Record #** | **Article Name {REF#]** | **Year** | **Number of SDOHs addressed** | **Type of SDOH addressed** | **Type of hospital organization** | **United States geographic region** | **Diseases, conditions, outcomes addressed** | **Services provided** |
| --- | --- | --- | --- | --- | --- | --- | --- | --- |
| **1** | Missed psychosocial risk factors during routine preoperative evaluations are associated with increased complications after elective cancer surgery [45]. | 2019 | 6 | Psychosocial risk factors including depression, smoking, alcohol, low resourcefulness | Academic Medical Center | Northeast | Diabetes, COPD, Heart failure, Liver disease, Disseminated cancer, Chronic steroid use, Weight loss, Bleeding disorder, Preoperative sepsis | Screening and Referral for Social Needs |
| **2** | Outpatient Palliative Care for Noncancer Illnesses: One Program's Experience with Implementation, Impact, and Lessons Learned [46]. | 2022 | 4 | Family Caregiver Support; psychosocial distress; spiritual needs; healthcare access and coordination | Academic Medical Center | West | Neurologic, Pulmonary, Cardiovascular, Gastrointestinal, Multimorbidity, Renal, Infectious/immunologic | Others (In-clinic care services) |
| **3** | Financial Incentives for Smoking Cessation in Hospitalized Patients: A Randomized Clinical Trial [34]. | 2020 | 1 | Low-income | Veteran's Affairs Health System | Northeast | Smoking | Financial Support Services (cost coverage) |
| **4** | Real-World Disparities in Remote Follow-Up of Cardiac Implantable Electronic Devices and Impact of the COVID-19 Pandemic: A Single-Center Experience [47]. | 2023 | 5 | Race/ethnicity, income, education, ancestry, spoken language | Academic Medical Center | Northeast | Cardiac conditions; people on pacemakers and other implanted devices | Digital Health Intervention (Remote monitoring services) |
| **5** | Implementing an EHR-based Screening and Referral System to Address Social Determinants of Health in Primary Care [48]. | 2019 | Multiple | Examples: Employment (12%), food insecurity (11%), and problems affording medications (11%) were the most prevalent concerns among respondents. | Academic Health Center | Northeast | Urban adult primary care | Screening and Referral for Social Needs |
| **6** | Documentation and review of social determinants of health data in the EHR: measures and associated insights [49]. | 2021 | Multiple | Housing, transportation, food insecurity, financial strain | Academic Medical Center | West | Not discussed | Not discussed |
| **7** | Effect of Community Health Workers on 30-Day Hospital Readmissions in an Accountable Care Organization Population: A Randomized Clinical Trial [50]. | 2021 | Multiple | Clinical access, access to social resources, assistance with transportation, psychosocial support, health coaching, using motivational interviewing, goal-setting, and other behavioral strategies. | Academic Medical Center | Northeast | Target population based on high risk for inpatient readmission | Community Health Worker Services |
| **8** | The impact of enhancing self-management support for diabetes in Community Health Centers through patient engagement and relationship building: a primary care pragmatic cluster-randomized trial [51]. | 2022 | 8 | Actionable social risks (e.g., food insecurity, housing instability); psychological outcomes (health distress, depression symptoms) | Community Health Center | West | Type 2 Diabetes | Community Health Worker Services (Care coordination and self-management support) |
| **9** | Online Telehealth Delivery of Group Mental Health Treatment Is Safe, Feasible, and Increases Enrollment and Attendance in Post-9/11 U.S. Veterans [52]. | 2022 | 3 | Social isolation; Social support; social cohesion | Veteran's Affairs Health System | Northeast | Post-Traumatic Stress Disorder, military reintegration | Digital Health Intervention (Tele mental health services) |
| **10** | Computerized Intervention in Primary Care for Women Veterans with Sexual Assault Histories and Psychosocial Health Risks: A Randomized Clinical Trial [53]. | 2022 | 3 | Psychosocial health risks (primary outcome) | Veteran's Affairs Health System | Southwest | Sexual Assault Survivorship | Digital Health Intervention (Health education mobile application) |
| **11** | Bridge to Health/Puente a la Salud: a pilot randomized trial to address diabetes self-management and social needs among high-risk patients [54]. | 2022 | 4 | Insufficient housing; Financial hardship; Food insecurity; Lack of transportation | Nonprofit community health system | Southwest | Type 2 Diabetes | Community Health Worker Services (Navigation Support) |
| **12** | Impact of a Low-Intensity Resource Referral Intervention on Patients' Knowledge, Beliefs, and Use of Community Resources: Results from the Community Rx Trial [55]. | 2020 | 5 | social, environmental, and behavioral conditions outside the healthcare system: income; housing insecurity; food insecurity; health behaviors, including diet, physical activity, and smoking | Academic Medical Center | Midwest | Depression, Diabetes, Smoking, Hypertension, Obesity | Digital Health Intervention |
| **13** | Patient navigation to address sociolegal barriers for patients with cancer: A comparative-effectiveness study [56]. | 2022 | 5 | Socio-legal barriers and language barriers | Safety-net medical center | Northeast | Lung cancer, breast cancer | Community Health Worker Services (Enhanced Navigation Support) |
| **14** | Nurse-led hospital-to-community care, clinical outcomes for people living with HIV and health-related social needs [57]. | 2022 | Multiple Health Related Social Needs | Education; Unemployed; Transportation; Race/Ethnicity; Gender; Housing; Insurance; Mental Health; Substance Use Disorder | Academic Medical Center | Northeast | HIV | Community Health Worker Services (Transitional care) |
| **15** | Improving Diabetes Care Through Population Health Innovations and Payments: Lessons from Western Maryland [58]. | 2023 | Multiple | Unmet social needs; food housing, transportation, etc. | Academic Medical Center | Northeast | Diabetes | Community Health Worker Services (Care coordination and self-management support) |
| **16** | Financing Diabetes Care in the U.S. Health System: Payment Innovations for Addressing the Medical and Social Determinants of Health [59]. | 2019 | 1 | Health Insurance | Nonprofit community hospitals and health systems | Not specified | Diabetes | Community Health Worker Services (Self-management support) |
| **17** | Clinical and Socio-behavioral Prediction Model of 30-Day Hospital Readmissions Among People with HIV and Substance Use Disorder: Beyond Electronic Health Record Data [60]. | 2019 | 2 | Food insecurity and readiness for substance use treatment | Safety-net medical center | Multiple geographic regions | HIV (Human Immunodeficiency Virus, Substance Use Disorder | Others (Validation of prediction models) |
| **18** | Effects of a standardized community health worker intervention on hospitalization among disadvantaged patients with multiple chronic conditions: A pooled analysis of three clinical trials [61]. | 2020 | 4 | Food insecurity, housing instability, drug and alcohol use, and presence or absence of social support | Academic Health Center | Northeast | Multiple chronic conditions: hypertension; diabetes; obesity; tobacco | Community Health Worker Services  (Social support) |
| **19** | Screening for health-related social needs in the emergency department: Adaptability and fidelity during the COVID-19 pandemic [43]. | 2022 | 5 | Food insecurity, Housing instability, Transportation needs, Utility assistance, and Interpersonal safety | Safety-net medical center | West | Not specified | Screening and Referral for Social Needs |
| **20** | Food Insecurity in Older Adults in an Integrated Health Care System [62]. | 2018 | 1 | Food insecurity | Nonprofit Community Health System (Integrated) | West | Food insecurity | Others (Analysis of food insecurity analysis) |
| **21** | Development and Implementation of a Maryland State Program Providing Hospital Payment Incentives for Reduction in Readmission Disparities [63]. | 2023 | 3 | Medicaid coverage, Race, and Area Deprivation Index | Nonprofit Community Health System | Northeast | Acute myocardial infarction, congestive heart failure, pneumonia | Financial Support Services (Financial incentives to reduce disparities) |
| **22** | Implementation fidelity to a behavioral diabetes prevention intervention in two New York City safety net primary care practices [64]. | 2023 | 3 | Social needs to effectively self-manage diabetes; | Veterans Affairs | Northeast | Diabetes | Community Health Worker Services (Outreach and coaching intervention) |
| **23** | Implementing Centering Pregnancy Group Prenatal Care for Minority Women Living with HIV at an Urban University Hospital [65]. | 2019 | 2 | HIV Diagnosis and Healthcare Access and Social Support | Academic Health Center | Southeast | HIV in pregnant women | Others (In-clinic care services) |
| **24** | Management of Isolated Skull Fractures in Pediatric Patients: A Systematic Review [66]. | 2019 | 3 | Income and Socioeconomic Status, Education, and Access to Healthcare | Academic Health Centers | Not specified | Isolated linear skull fractures in pediatric patients | Community Health Worker Services (Transitional care) |
| **25** | Health Center-Based Community-Supported Agriculture: An RCT [67]. | 2019 | 11 | Food insecurity; patient-reported psychosocial outcomes | Community Health Center | Northeast | Body Mass Index; eating behaviors | Community Health Worker Services (Community support agriculture) |
| **26** | Effects of In-Person Assistance vs Personalized Written Resources About Social Services on Household Social Risks and Child and Caregiver Health: A Randomized Clinical Trial [68]. | 2020 | 18 | Childhood social and economic risk factors | Safety net hospital | West | Household social risk | Community Health Worker Services (Navigation support) |
| **27** | Feasibility of a family-centered intervention for depressed older men in primary care [44]. | 2019 | 3 | Family support; Language; Ethnicity | Federally Qualified Health Center | West | Depression | Others (In-clinic care services) |
| **28** | Intensive care management for high-risk veterans in a patient-centered medical home - do some veterans benefit more than others? [69]. | 2023 | 6+ | Travel distance, copay exemption, risk score for future hospitalizations, history of hospital discharge against medical advice, homelessness, and multiple residence ZIP codes. | Veteran Affairs Healthcare System | West | High-cost healthcare utilization | Others (In-clinic care services) |
| **29** | Pragmatic Clinical Trial to Improve Patient Experience Among Adults During Transitions from Hospital to Home: The PArTNER study [70]. | 2022 | 3 | Social health & social support measures: emotional, instrumental, and informational support | Minority Serving Hospital | Midwestern | Healthcare utilization; emotional, social, and physical health at 30 days post discharge | Community Health Worker Services (Navigator intervention vs usual care) |
| **30** | Medical-Legal Partnership Effects on Mental Health, Health Care Use, and Quality of Life in Primary Care: A Randomized Clinical Trial [71]. | 2023 | 1 | Health Harming Legal Needs (HHLN) | Academic Medical Center; | Southwest | Mental health, health care use, and quality of life | Screening and Referral for Social Needs |
| **31** | Predictive Model-Driven Hot spotting to Decrease Emergency Department Visits: A Randomized Controlled Trial [72]. | 2021 | Multiple | Social needs that may place adults at high risk for ED utilization, including food and housing insecurity | Academic Medical Center | Midwest | High risk healthcare utilization; ED visits and inpatient admissions | Community Health Worker Services (Community based and coordinated case management) |
| **32** | A care coordinator screening strategy to address health harming legal needs [73]. | 2022 | 8 | Solutions for Health Harming Legal Needs | Safety net healthcare system | Midwest | Not discussed | Others (Legal assistance) |
| **33** | Evidence that collaborative action between local health departments and nonprofit hospitals helps foster healthy behaviors in communities: a multilevel study [74]. | 2021 | 1 | Community Health Needs | Nonprofit Community Hospitals | Multiple geographic regions | Healthy eating and lifestyle habits | Not discussed |
| **34** | Hospital Partnerships for Population Health: A Systematic Review of the Literature [23]. | 2021 | 4 | Community health needs assessment, care coordination/transition, health and wellness, public health activities | Academic Heath Centers and Health Systems | Not specified | Not discussed | Not discussed |
| **35** | Medicaid Investments to Address Social Needs in Oregon and California [75]. | 2019 | 4 | Care coordination, Housing, Food insecurity, Legal needs | Coordinated Care Organizations | West | No specific disease or condition | Financial support services (Alternative payment methods) |
| **36** | Enabling Services Improve Access to Care, Preventive Services, And Satisfaction Among Health Center Patients [76]. | 2019 | 6 | race, age, ethnicity, English proficiency, education, income, and mental health | Safety net health center | National | COPD, CVD, Obesity, Asthma, Diabetes, Mental health | Others (Improved access) |
| **37** | A Social-Return-On-Investment Analysis of Bon Secours Hospital's 'Housing for Health' Affordable Housing Program [77]. | 2021 | 3 | Economic stability, Neighborhood and Built Environment (Affordable housing), Social and Community Context | Safety net hospital | Northeast | No specific disease or condition | Financial Support Services (Affordable housing) |
| **38** | Embedding Social Workers in Veterans Health Administration Primary Care Teams Reduces Emergency Department Visits [78]. | 2020 | 1 | Care coordination for high-risk, high-need populations | Veteran’s Affairs | Multiple geographic regions | Hospital admissions, Emergency department visits, social work encounters | Community Health Worker Services |
| **39** | Project Nurture Integrates Care and Services to Improve Outcomes for Opioid-Dependent Mothers and Their Children [79]. | 2020 | 3 | Integration of care and services, coordination between sectors, and Improving outcomes for opioid-dependent mothers and their children | Coordinated care organization | West | Range of patient and child welfare outcomes, including child maltreatment and foster care placement | Community Health Worker Services (Care coordination and navigation support) |
| **40** | Addressing the Poverty Barrier in Collaborative Care for Adults Experiencing Homelessness: A Case-Based Report [80]. | 2020 | 3 | Homelessness, Food insecurity, Unemployment | Federally Qualified Health Center | Southeast | Depression and Homelessness | Others (Behavioral and educational intervention) |
| **41** | Interprofessional, multitiered daily rounding management in a high-acuity hospital [81]. | 2020 | Not specified | Not specified | Academic Medical Center | Northeast | Length of Stay, Subjective Symptom Expressions, Healthcare Associated Infections, hospital-acquired pressure injuries as well as improved patient experience | Others (Lean Daily Management) |
| **42** | Indianapolis Provider's Use of Wraparound Services Associated with Reduced Hospitalizations and Emergency Department Visits [82]. | 2018 | 5 | Mental health needs, Nutritional needs, Access to care, social issues affecting health, healthcare spending | Safety net hospital | Midwest | Social, behavioral, and environmental factors that drive poor patient health and increase costs, Number of subsequent hospitalizations and ED visits | Others (Wraparound services) |
| **43** | Reasons for Social Work Referrals in an Urban Safety-Net Population: A Natural Language Processing and Market Basket Analysis Approach [83]. | 2021 | 17 | Financial, Food/Food Insecurity, Violence and Safety, Housing, Legal, Transportation, Behavioral Health, Aging, Education, Employment, Family/Social Support, Pregnancy, Language services, Disability, Community Resources, Adherence to treatment | Federally Qualified Health Center | Midwest | No specific disease or condition | Screening and Referral for Social Needs |
| **44** | The Impact of Food Insecurity Screenings and Community Food Resource Referrals for Patients with Type 2 Diabetes [84]. | 2020 | 1 | Food Insecurity | Federally Qualified Health Center | West | Type 2 diabetes | Screening and Referral for Social Needs |
| **45** | The implementation cost of a safety‐net hospital program addressing social needs in Atlanta [85]. | 2021 | Multiple | Social needs including social support, housing, financial strain and problems, and transportation.  (Basically, socioeconomic barriers to treatment plan adherence) | Safety Net Hospital | Southeast | Heart Failure | Community Health Worker Services |
| **46** | Development of a homelessness risk screening tool for emergency department patients [86]. | 2022 | 1 | Homelessness | Safety Net Hospital | Northeast | No specific disease or condition | Others (Development of screening tool for future risk prediction) |
| **47** | An embedded multiple case study: using CFIR to map clinical food security screening constructs for the development of primary care practice guidelines [87]. | 2022 | 1 | Food Insecurity | Academic Medical Center | Midwest | No specific disease or condition | Screening and Referral for Social Needs |
| **48** | Conceptualizing the effective mechanisms of a social needs case management program shown to reduce hospital use: a qualitative study [88]. | 2022 | Multiple | Social needs such as housing, food, transportation, and income | Public health department (Contra Costa) | West | High risk of acute care utilization | Others (Wraparound services) |
| **49** | Social Needs Screening and Referral Program at a Large US Public Hospital System, 2017 [89]. | 2020 | 8 | Food insecurity, health insurance coverage, housing concerns, public income benefits, household interpersonal violence, adult education and literacy, daycare, and general & immigration legal problems) | Safety net hospital | Northeast | Not specified | Screening and Referral for Social Needs |
| **50** | A Nurse-Led Intervention to Address Food Insecurity in Chicago [90]. | 2018 | 2 | Food Insecurity and Environmental Waste (Food waste) | Academic Medical Center | Midwest | Not specified | Community Health Worker Services (Meal distribution) |
| **51** | Training Student Volunteers as "Community Resource Navigators" to Integrate Health and Social Care in Primary Care [91]. | 2022 | Multiple | Unmet social needs such as food, transportation, housing identified using the PREPARE screening tool | Federally Qualified Health Center | Southeast | No specific disease or condition or behaviors addressed | Screening and Referral for Social Needs |
| **52** | An In-Clinic Food Pharmacy Addresses Very Low Food Security [92]. | 2020 | 1 | Food Insecurity | Nonprofit health system (Atrium Health) | Southeast | No specific disease or condition or behaviors addressed | Screening and Referral for Social Needs |
| **53** | Evidence based processes to prevent readmissions: more is better, a ten-site observational study [93]. | 2021 | Multiple | Unmet social needs; food housing, transportation, etc. | Veterans Affairs | Southwest | Chronic conditions; High risk for readmission | Community Health Worker Services (Transitional care) |
| **54** | Unmet Social Needs and No-Show Visits in Primary Care in a US Northeastern Urban Health System, 2018–2019 [94]. | 2020 | Multiple | Unmet social needs; food housing, transportation, etc. | Nonprofit community hospital or health system | Northeast | No-shows | Screening and Referral for Social Needs |
| **55** | Cooling The Hot Spots Where Child Hospitalization Rates Are High: A Neighborhood Approach to Population Health [95]. | 2019 | Multiple | Social risk | Children's medical center | Midwest | Child hospitalization rates | Community Health Worker Services (Transitional care) |
| **56** | Quantifying Health Systems' Investment in Social Determinants of Health, By Sector, 2017–19 [96]. | 2020 | 6 | Housing, food, employment, transportation, education, social and community context | Nonprofit community hospital or health system | National | Housing insecurity; food insecurity; transportation barriers | Financial Support Services (Financial investment in social determinants of health) |
| **57** | Prescribing Housing: A Scoping Review of Health System Efforts to Address Housing as a Social Determinant of Health [34]. | 2021 | 1 | Housing | Nonprofit community hospital or health system | National | Housing instability | Financial Support Services (Financial investment in housing) |
| **58** | The Baltimore Community-Based Organizations Neighborhood Network: Enhancing Capacity Together (CONNECT) Cluster RCT [97]. | 2019 | Multiple | Housing, food, transportation, etc. | Academic medical center | Northeast | Healthcare use | Screening and Referral for Social Needs |
| **59** | Effect of Community Health Worker Support on Clinical Outcomes of Low-Income Patients Across Primary Care Facilities: A Randomized Clinical Trial [98]. | 2018 | Multiple | Health literacy, unmet social need, social support, etc. | Nonprofit community hospital or health system | Northeast | Self-health rating; perceived quality of care; healthcare utilization | Community Health Worker Services (Individualized goal setting and case management) |
| **60** | Food for Thought: A Randomized Trial of Food Insecurity Screening in the Emergency Department [99]. | 2019 | 1 | Food Insecurity | Children's Hospital | Northeast | Food insecurity screening | Screening and Referral for Social Needs |
| **61** | Effect of a Peer-Led Behavioral Intervention for Emergency Department Patients at High Risk of Fatal Opioid Overdose: A Randomized Clinical Trial [100]. | 2022 | 1 | Opioid Overdose | Academic Health Center | Northeast | Substance use disorder | Not discussed |
| **62** | Patient plus partner trial: A randomized controlled trial of 2 interventions to improve outcomes after an initial implantable cardioverter-defibrillator [101]. | 2019 | Not Specified | Not Specified | Nonprofit community hospital or health system | Southwest | Cardiovascular diseases, anxiety, and depression | Not discussed |
| **63** | Illustrating the value of social work: results of an open pilot trial of the psychosocial acuity scale in a large urban pediatric hospital [102]. | 2021 | 1 | Psychosocial risk | Children's hospital | Northeast | Psychosocial risks documentation in medical record | Community Health Worker Services (Improving social work documentation) |
| **64** | Using Publicly Available Data to Understand the Opioid Overdose Epidemic: Geospatial Distribution of Discarded Needles in Boston, Massachusetts [103]. | 2018 | Not documented | Not documented | Academic Medical Center | Northeast | Opioid overdose | Others (Hot spotting) |
| **65** | Predictors for Poor Linkage to Care Among Hospitalized Persons Living with HIV and Co-Occurring Substance Use Disorder [104]. | 2020 | Multiple | Education, Housing, Insurance | Academic Medical Center | Southeast | HIV | Community Health Worker Services (Navigation support) |
| **66** | Enhanced care planning and clinical-community linkages versus usual care to address basic needs of patients with multiple chronic conditions: a clinician-level randomized controlled trial [105]. | 2020 | Multiple | Health Related Social Needs | Academic Medical Center | Southeast | MCC | Community Health Worker Services (Navigation support) |
| **67** | Social Risks Among Primary Care Patients in a Large Urban Health System [27]. | 2020 | 10 | Quality Housing, Instability, Access to healthcare, Food insecurity, Health travel, Health cost, Getting along, utilities cost, care need, legal, Interpersonal violence | Federally Qualified Health Centers | Northeast | Not specified | Screening and Referral for Social Needs |
| **68** | Relationship of Neighborhood Deprivation and Outcomes of a Comprehensive ST-Segment-Elevation Myocardial Infarction Protocol [106]. | 2021 | Not specified | Not specified | Academic Medical Center | Midwest | Door-to-balloon time (D2BT), In-hospital mortality, Use of guideline-directed medical therapy (GDMT) before percutaneous coronary intervention (PCI) and the adoption of trans radial access for PCI. | Others (In-clinic care services) |
| **69** | Enhancing Reach Out and Read with a Video and Text Messages: A Randomized Trial in a Low-Income Predominantly Latino Sample [107]. | 2021 | 1 | Early Literacy | Federally Qualified Health Center | Northeast | Low-income background | Others (Behavioral and educational Intervention) |
| **70** | Social Needs Screening in Hospitalized Pediatric Patients: A Randomized Controlled Trial [108]. | 2023 | Multiple | Individual-level adverse Social Determinants | Children's hospital | Southwest | Not specified | Screening and Referral for Social Needs |

**Table 3: Data Charting in Response to RQ #3 (Emphasis of Initiatives) and RQ#4 (EHR Use to Integrate SDOH Data into Care Practices)**

| **Record #** | **Article Name {REF#]** | **Upstream initiative vs Downstream initiative to address SDOH** | **Screening & Referral VS. Neighborhood Hot spotting** | **Quality Improvement (QI) VS. Population Health Management (PHM)** | **Integration of SDOH data into care practices through the EHR** | | | | |
| --- | --- | --- | --- | --- | --- | --- | --- | --- | --- |
|  |  |  |  |  | **Step 1: Collected/organized patient-reported or community-level SDOH data in the EHR** | **Step 2: Integrated SDOH data into care workflows using the EHR.** | | **Step 3: Developed EHR-based automated support and action based on SDOH data** | **Step 4: Evaluated the impact of integrating SDOH into care delivery** |
|  |  |  |  |  |  | **Were patient-level SDOH data shared with the care team?** | **Did the care team discuss SDOH results with patients?** |  |  |
| **1** | Missed psychosocial risk factors during routine preoperative evaluations are associated with increased complications after elective cancer surgery [45]. | Downstream | Screening and Referral | Quality Improvement | Yes | Not documented | Not documented | Not documented | Yes |
| **2** | Outpatient Palliative Care for Noncancer Illnesses: One Program's Experience with Implementation, Impact, and Lessons Learned [46]. | Downstream | Hot spotting | Quality Improvement | Yes | Yes | Yes | Yes | Yes |
| **3** | Financial Incentives for Smoking Cessation in Hospitalized Patients: A Randomized Clinical Trial [34]. | Downstream | Hot spotting | Mixed | Not documented | Not documented | Not documented | Not documented | Yes |
| **4** | Real-World Disparities in Remote Follow-Up of Cardiac Implantable Electronic Devices and Impact of the COVID-19 Pandemic: A Single-Center Experience [47]. | Downstream | Hot spotting | Mixed | Yes | Not documented | Not documented | Not documented | Not documented |
| **5** | Implementing an EHR-based Screening and Referral System to Address Social Determinants of Health in Primary Care [48]. | Downstream | Screening and Referral | Quality Improvement | Yes | Yes | Yes | Yes | Yes |
| **6** | Documentation and review of social determinants of health data in the EHR: measures and associated insights [49]. | Downstream | Screening and Referral | Quality Improvement | Yes | Not documented | Not documented | Not documented | Not documented |
| **7** | Effect of Community Health Workers on 30-Day Hospital Readmissions in an Accountable Care Organization Population: A Randomized Clinical Trial [50]. | Downstream | Hot spotting | Population Health Management | Yes | Yes | Not documented | Not documented | Yes |
| **8** | The impact of enhancing self-management support for diabetes in Community Health Centers through patient engagement and relationship building: a primary care pragmatic cluster-randomized trial [51]. | Downstream | Hot spotting | Mixed | Not documented | Yes | Yes | Not documented | Yes |
| **9** | Online Telehealth Delivery of Group Mental Health Treatment Is Safe, Feasible, and Increases Enrollment and Attendance in Post-9/11 U.S. Veterans [52]. | Downstream | Hot spotting | Mixed | Not documented | Yes | Yes | Not documented | Yes |
| **10** | Computerized Intervention in Primary Care for Women Veterans with Sexual Assault Histories and Psychosocial Health Risks: A Randomized Clinical Trial [53]. | Downstream | Hot spotting | Mixed | Not documented | Not documented | Not documented | Not documented | Yes |
| **11** | Bridge to Health/Puente a la Salud: a pilot randomized trial to address diabetes self-management and social needs among high-risk patients [54]. | Mixed | Hot spotting | Mixed | Not documented | Not documented | Not documented | Not documented | Yes |
| **12** | Impact of a Low-Intensity Resource Referral Intervention on Patients' Knowledge, Beliefs, and Use of Community Resources: Results from the Community Rx Trial [55]. | Downstream | Screening and Referral | Population Health Management | Yes | Yes | Yes | Yes | Not documented |
| **13** | Patient navigation to address sociolegal barriers for patients with cancer: A comparative-effectiveness study [56]. | Downstream | Screening and Referral | Quality Improvement | Not documented | Yes | Yes | Not documented | Yes |
| **14** | Nurse-led hospital-to-community care, clinical outcomes for people living with HIV and health-related social needs [57]. | Downstream | Screening and Referral | Mixed | Yes | Yes | Yes | Not documented | Yes |
| **15** | Improving Diabetes Care Through Population Health Innovations and Payments: Lessons from Western Maryland [58]. | Mixed | Hot spotting | Mixed | Yes | Yes | Yes | Not documented | Yes |
| **16** | Financing Diabetes Care in the U.S. Health System: Payment Innovations for Addressing the Medical and Social Determinants of Health [59]. | Mixed | Hot spotting | Population Health Management | Not documented | Not documented | Not documented | Not documented | Not documented |
| **17** | Clinical and Socio-behavioral Prediction Model of 30-Day Hospital Readmissions Among People with HIV and Substance Use Disorder: Beyond Electronic Health Record Data [60]. | Downstream | Hot spotting | Population Health Management | Yes | Not documented | Not documented | Not documented | Yes |
| **18** | Effects of a standardized community health worker intervention on hospitalization among disadvantaged patients with multiple chronic conditions: A pooled analysis of three clinical trials [61]. | Downstream | Hot spotting | Population Health Management | Not documented | Not documented | Not documented | Not documented | Yes |
| **19** | Screening for health-related social needs in the emergency department: Adaptability and fidelity during the COVID-19 pandemic [43]. | Downstream | Screening and Referral | Quality Improvement | Yes | Not documented | Not documented | Not documented | Not documented |
| **20** | Food Insecurity in Older Adults in an Integrated Health Care System [62]. | Mixed | Hot spotting | Population Health Management | Not documented | Not documented | Not documented | Not documented | Not documented |
| **21** | Development and Implementation of a Maryland State Program Providing Hospital Payment Incentives for Reduction in Readmission Disparities [63]. | Downstream | Hot spotting | Mixed | Not documented | Not documented | Not documented | Not documented | Not documented |
| **22** | Implementation fidelity to a behavioral diabetes prevention intervention in two New York City safety net primary care practices [64]. | Downstream | Hot spotting | Mixed | Not documented | Not documented | Yes | Not documented | Yes |
| **23** | Implementing Centering Pregnancy Group Prenatal Care for Minority Women Living with HIV at an Urban University Hospital [65]. | Downstream | Hot spotting | Mixed | Yes | Yes | Not documented | Not documented | Not documented |
| **24** | Management of Isolated Skull Fractures in Pediatric Patients: A Systematic Review [66]. | Downstream | Hot spotting | Quality Improvement | Not documented | Yes | Not documented | Not documented | Not documented |
| **25** | Health Center-Based Community-Supported Agriculture: An RCT [67]. | Upstream | Hot spotting | Population Health Management | Not documented | Not documented | Not documented | Not documented | Yes |
| **26** | Effects of In-Person Assistance vs Personalized Written Resources About Social Services on Household Social Risks and Child and Caregiver Health: A Randomized Clinical Trial [68]. | Downstream | Hot spotting | Population Health Management | Not documented | Not documented | Not documented | Not documented | Yes |
| **27** | Feasibility of a family-centered intervention for depressed older men in primary care [44]. | Downstream | Hot spotting | Population Health Management | Not documented | Not documented | Not documented | Not documented | Yes |
| **28** | Intensive care management for high-risk veterans in a patient-centered medical home - do some veterans benefit more than others? [69]. | Downstream | Hot spotting | Mixed | Not documented | Not documented | Not documented | Not documented | Yes |
| **29** | Pragmatic Clinical Trial to Improve Patient Experience Among Adults During Transitions from Hospital to Home: The PArTNER study [70]. | Downstream | Hot spotting | Quality Improvement | Not documented | Yes | Yes | Not documented | Yes |
| **30** | Medical-Legal Partnership Effects on Mental Health, Health Care Use, and Quality of Life in Primary Care: A Randomized Clinical Trial [71]. | Mixed | Hot spotting | Population Health Management | Not documented | Yes | Not documented | Not documented | Yes |
| **31** | Predictive Model-Driven Hot spotting to Decrease Emergency Department Visits: A Randomized Controlled Trial [72]. | Downstream | Hot spotting | Population Health Management | Not documented | Yes | Yes | Not documented | Yes |
| **32** | A care coordinator screening strategy to address health harming legal needs [73]. | Downstream | Screening and Referral | Population Health Management | Not documented | Yes | Not documented | Not documented | Yes |
| **33** | Evidence that collaborative action between local health departments and nonprofit hospitals helps foster healthy behaviors in communities: a multilevel study [74]. | Upstream | Hot spotting | Population Health Management | Not documented | Not documented | Not documented | Not documented | Yes |
| **34** | Hospital Partnerships for Population Health: A Systematic Review of the Literature [23]. | Upstream | Hot spotting | Population Health Management | Not documented | Not documented | Not documented | Not documented | Yes |
| **35** | Medicaid Investments to Address Social Needs in Oregon and California [75]. | Upstream | Hot spotting | Population Health Management | Not documented | Not documented | Not documented | Not documented | Yes |
| **36** | Enabling Services Improve Access to Care, Preventive Services, And Satisfaction Among Health Center Patients [76]. | Mixed | Hot spotting | Population Health Management | Not documented | Not documented | Not documented | Not documented | Yes |
| **37** | A Social-Return-On-Investment Analysis of Bon Secours Hospital's 'Housing for Health' Affordable Housing Program [77]. | Upstream | Hot spotting | Population Health Management | Not documented | Not documented | Not documented | Not documented | Not documented |
| **38** | Embedding Social Workers in Veterans Health Administration Primary Care Teams Reduces Emergency Department Visits [78]. | Downstream | Hot spotting | Mixed | Yes | Yes | Not documented | Not documented | Yes |
| **39** | Project Nurture Integrates Care and Services to Improve Outcomes for Opioid-Dependent Mothers and Their Children [79]. | Downstream | Hot spotting | Mixed | Not documented | Yes | Not documented | Not documented | Yes |
| **40** | Addressing the Poverty Barrier in Collaborative Care for Adults Experiencing Homelessness: A Case-Based Report [80]. | Mixed | Hot spotting | Population Health Management | Not documented | Yes | Yes | Not documented | Yes |
| **41** | Interprofessional, multitiered daily rounding management in a high-acuity hospital [81]. | Downstream | Hot spotting | Mixed | Not documented | Not documented | Not documented | Not documented | Not documented |
| **42** | Indianapolis Provider's Use of Wraparound Services Associated with Reduced Hospitalizations and Emergency Department Visits [82]. | Downstream | Hot spotting | Mixed | Yes | Yes | Not documented | Not documented | Yes |
| **43** | Reasons for Social Work Referrals in an Urban Safety-Net Population: A Natural Language Processing and Market Basket Analysis Approach [83]. | Downstream | Screening and Referral | Population Health Management | Yes | Yes | Yes | Not documented | Not documented |
| **44** | The Impact of Food Insecurity Screenings and Community Food Resource Referrals for Patients with Type 2 Diabetes [84]. | Downstream | Screening and Referral | Mixed | Yes | Yes | Yes | Not documented | Yes |
| **45** | The implementation cost of a safety‐net hospital program addressing social needs in Atlanta [85]. | Downstream | Screening and Referral | Population Health Management | Not documented | Yes | Yes | Not documented | Yes |
| **46** | Development of a homelessness risk screening tool for emergency department patients [86]. | Downstream | Screening and Referral | Quality Improvement | Not documented | Not documented | Not documented | Not documented | Not documented |
| **47** | An embedded multiple case study: using CFIR to map clinical food security screening constructs for the development of primary care practice guidelines [87]. | Mixed | Screening and Referral | Quality Improvement | Yes | Yes | Yes | Not documented | Yes |
| **48** | Conceptualizing the effective mechanisms of a social needs case management program shown to reduce hospital use: a qualitative study [88]. | Downstream | Hot spotting | Population Health Management | Yes | Yes | Yes | Not documented | Yes |
| **49** | Social Needs Screening and Referral Program at a Large US Public Hospital System, 2017 [89]. | Downstream | Screening and Referral | Mixed | Yes | Yes | Yes | Not documented | Not documented |
| **50** | A Nurse-Led Intervention to Address Food Insecurity in Chicago [90]. | Downstream | Hot spotting | Population Health Management | Not documented | Not documented | Not documented | Not documented | Yes |
| **51** | Training Student Volunteers as "Community Resource Navigators" to Integrate Health and Social Care in Primary Care [91]. | Downstream | Hot spotting | Population Health Management | Not documented | Not documented | Not documented | Not documented | Yes |
| **52** | An In-Clinic Food Pharmacy Addresses Very Low Food Security [92]. | Downstream | Hot spotting | Mixed | Yes | Yes | Yes | Not documented | Not documented |
| **53** | Evidence based processes to prevent readmissions: more is better, a ten-site observational study [93]. | Downstream | Hot spotting | Mixed | Not documented | Not documented | Not documented | Not documented | Yes |
| **54** | Unmet Social Needs and No-Show Visits in Primary Care in a US Northeastern Urban Health System, 2018–2019 [94]. | Downstream | Screening and Referral | Mixed | Yes | Yes | Yes | Not documented | Yes |
| **55** | Cooling The Hot Spots Where Child Hospitalization Rates Are High: A Neighborhood Approach to Population Health [95]. | Downstream | Hot spotting | Population Health Management | Yes | Yes | Yes | Not documented | Yes |
| **56** | Quantifying Health Systems' Investment in Social Determinants of Health, By Sector, 2017–19 [96]. | Upstream | Hot spotting | Population Health Management | Not documented | Not documented | Not documented | Not documented | Not documented |
| **57** | Prescribing Housing: A Scoping Review of Health System Efforts to Address Housing as a Social Determinant of Health [34]. | Mixed | Hot spotting | Population Health Management | Not documented | Not documented | Not documented | Not documented | Not documented |
| **58** | The Baltimore Community-Based Organizations Neighborhood Network: Enhancing Capacity Together (CONNECT) Cluster RCT [97]. | Mixed | Hot spotting | Population Health Management | Not documented | Not documented | Not documented | Not documented | Not documented |
| **59** | Effect of Community Health Worker Support on Clinical Outcomes of Low-Income Patients Across Primary Care Facilities: A Randomized Clinical Trial [98]. | Downstream | Hot spotting | Mixed | Not documented | Not documented | Not documented | Not documented | Not documented |
| **60** | Food for Thought: A Randomized Trial of Food Insecurity Screening in the Emergency Department [99]. | Downstream | Screening and Referral | Mixed | Not documented | Not documented | Not documented | Not documented | Not documented |
| **61** | Effect of a Peer-Led Behavioral Intervention for Emergency Department Patients at High Risk of Fatal Opioid Overdose: A Randomized Clinical Trial [100]. | Downstream | Hot spotting | Mixed | Not documented | Not documented | Not documented | Not documented | Not documented |
| **62** | Patient plus partner trial: A randomized controlled trial of 2 interventions to improve outcomes after an initial implantable cardioverter-defibrillator [101]. | Downstream | Hot spotting | Quality Improvement | Not documented | Not documented | Not documented | Not documented | Not documented |
| **63** | Illustrating the value of social work: results of an open pilot trial of the psychosocial acuity scale in a large urban pediatric hospital [102]. | Downstream | Screening and Referral | Quality Improvement | Yes | Yes | Not documented | Not documented | Not documented |
| **64** | Using Publicly Available Data to Understand the Opioid Overdose Epidemic: Geospatial Distribution of Discarded Needles in Boston, Massachusetts [103]. | Downstream | Hot spotting | Population Health Management | Not documented | Not documented | Not documented | Not documented | Not documented |
| **65** | Predictors for Poor Linkage to Care Among Hospitalized Persons Living with HIV and Co-Occurring Substance Use Disorder [104]. | Downstream | Hot spotting | Mixed | Not documented | Not documented | Not documented | Not documented | Not documented |
| **66** | Enhanced care planning and clinical-community linkages versus usual care to address basic needs of patients with multiple chronic conditions: a clinician-level randomized controlled trial [105]. | Downstream | Hot spotting | Mixed | Not documented | Not documented | Not documented | Not documented | Not documented |
| **67** | Social Risks Among Primary Care Patients in a Large Urban Health System [27]. | Mixed | Hot spotting | Population Health Management | Yes | Yes | Yes | Not documented | Not documented |
| **68** | Relationship of Neighborhood Deprivation and Outcomes of a Comprehensive ST-Segment-Elevation Myocardial Infarction Protocol [106]. | Mixed | Hot spotting | Mixed | Not documented | Yes | Not documented | Not documented | Not documented |
| **69** | Enhancing Reach Out and Read with a Video and Text Messages: A Randomized Trial in a Low-Income Predominantly Latino Sample [107]. | Downstream | Hot spotting | Mixed | Not documented | Not documented | Not documented | Not documented | Yes |
| **70** | Social Needs Screening in Hospitalized Pediatric Patients: A Randomized Controlled Trial [108]. | Downstream | Screening and Referral | Mixed | Not documented | Not documented | Not documented | Not documented | Yes |

**Table 4: Data Charting in Response to RQ #3 (Emphasis of Initiatives) and RQ#5) Challenges Encountered**

| **Record #** | **Article Name {REF#]** | **Includes outcome measures of health equity and/or health disparities (YES/NO)** | **Indicate measures of health disparity or health equity used or N/A** | **Internal Capacity vs Community Partnerships to address SDOH** | **Challenges reported in addressing SDOH** |
| --- | --- | --- | --- | --- | --- |
| **1** | Missed psychosocial risk factors during routine preoperative evaluations are associated with increased complications after elective cancer surgery [45]. | No | Not applicable | Internal capacity | Implementation challenges (Limited awareness) |
| **2** | Outpatient Palliative Care for Noncancer Illnesses: One Program's Experience with Implementation, Impact, and Lessons Learned [46]. | No | Not applicable | Internal capacity | Not discussed |
| **3** | Financial Incentives for Smoking Cessation in Hospitalized Patients: A Randomized Clinical Trial [34]. | No | Not applicable | Mixed | Not discussed |
| **4** | Real-World Disparities in Remote Follow-Up of Cardiac Implantable Electronic Devices and Impact of the COVID-19 Pandemic: A Single-Center Experience [47]. | Yes | Disparities in use of RM by race, education, income, ancestry | Mixed | Not discussed |
| **5** | Implementing an EHR-based Screening and Referral System to Address Social Determinants of Health in Primary Care [48]. | No | Not applicable | Internal capacity | Organizational challenges (Limited institutional support) |
| **6** | Documentation and review of social determinants of health data in the EHR: measures and associated insights [49]. | No | Not applicable | Internal capacity | Not discussed |
| **7** | Effect of Community Health Workers on 30-Day Hospital Readmissions in an Accountable Care Organization Population: A Randomized Clinical Trial [50]. | No | Not applicable | Internal capacity | Not discussed |
| **8** | The impact of enhancing self-management support for diabetes in Community Health Centers through patient engagement and relationship building: a primary care pragmatic cluster-randomized trial [51]. | No | Not applicable | Mixed | Not discussed |
| **9** | Online Telehealth Delivery of Group Mental Health Treatment Is Safe, Feasible, and Increases Enrollment and Attendance in Post-9/11 U.S. Veterans [52]. | No | Not applicable | Internal capacity | Not discussed |
| **10** | Computerized Intervention in Primary Care for Women Veterans with Sexual Assault Histories and Psychosocial Health Risks: A Randomized Clinical Trial [53]. | No | Not applicable | Internal capacity | Not discussed |
| **11** | Bridge to Health/Puente a la Salud: a pilot randomized trial to address diabetes self-management and social needs among high-risk patients [54]. | No | Not applicable | Mixed | Not discussed |
| **12** | Impact of a Low-Intensity Resource Referral Intervention on Patients' Knowledge, Beliefs, and Use of Community Resources: Results from the Community Rx Trial [55]. | No | Not applicable | Mixed | Not discussed |
| **13** | Patient navigation to address sociolegal barriers for patients with cancer: A comparative-effectiveness study [56]. | No | Not applicable | Internal capacity | Implementation challenges (Socio-legal barriers) |
| **14** | Nurse-led hospital-to-community care, clinical outcomes for people living with HIV and health-related social needs [57]. | No | Not applicable | Internal capacity | Organizational challenges (limited documentation and reporting) |
| **15** | Improving Diabetes Care Through Population Health Innovations and Payments: Lessons from Western Maryland [58]. | No | Not applicable | Internal capacity | Not discussed |
| **16** | Financing Diabetes Care in the U.S. Health System: Payment Innovations for Addressing the Medical and Social Determinants of Health [59]. | No | Not applicable | Internal capacity | Implementation challenges (Service integration, funding, regulation) |
| **17** | Clinical and Socio-behavioral Prediction Model of 30-Day Hospital Readmissions Among People with HIV and Substance Use Disorder: Beyond Electronic Health Record Data [60]. | No | Not applicable | Internal capacity | Not discussed |
| **18** | Effects of a standardized community health worker intervention on hospitalization among disadvantaged patients with multiple chronic conditions: A pooled analysis of three clinical trials [61]. | No | Not applicable | Internal capacity | Implementation challenges (Existence of multiple SDOH impacting one another) |
| **19** | Screening for health-related social needs in the emergency department: Adaptability and fidelity during the COVID-19 pandemic [43]. | No | Not applicable | Internal capacity | Not discussed |
| **20** | Food Insecurity in Older Adults in an Integrated Health Care System [62]. | No | Not applicable | Mixed | Not discussed |
| **21** | Development and Implementation of a Maryland State Program Providing Hospital Payment Incentives for Reduction in Readmission Disparities [63]. | Yes | Hospital-level disparity in readmission | Internal capacity | Not discussed |
| **22** | Implementation fidelity to a behavioral diabetes prevention intervention in two New York City safety net primary care practices [64]. | No | Not applicable | Mixed | Not discussed |
| **23** | Implementing Centering Pregnancy Group Prenatal Care for Minority Women Living with HIV at an Urban University Hospital [65]. | No | Not applicable | Internal capacity | Not discussed |
| **24** | Management of Isolated Skull Fractures in Pediatric Patients: A Systematic Review [66]. | Yes | Not specified | Internal capacity | Not discussed |
| **25** | Health Center-Based Community-Supported Agriculture: An RCT [67]. | No | Not applicable | Community Partnerships | Not discussed |
| **26** | Effects of In-Person Assistance vs Personalized Written Resources About Social Services on Household Social Risks and Child and Caregiver Health: A Randomized Clinical Trial [68]. | No | Not applicable | Mixed | Not discussed |
| **27** | Feasibility of a family-centered intervention for depressed older men in primary care [44]. | No | Not applicable | Mixed | Not discussed |
| **28** | Intensive care management for high-risk veterans in a patient-centered medical home - do some veterans benefit more than others? [69]. | No | Not applicable Not applicable | Internal capacity | Not discussed |
| **29** | Pragmatic Clinical Trial to Improve Patient Experience Among Adults During Transitions from Hospital to Home: The PArTNER study [70]. | No | Not applicable | Mixed | Not discussed |
| **30** | Medical-Legal Partnership Effects on Mental Health, Health Care Use, and Quality of Life in Primary Care: A Randomized Clinical Trial [71]. | No | Not applicable | Mixed | Not discussed |
| **31** | Predictive Model-Driven Hot spotting to Decrease Emergency Department Visits: A Randomized Controlled Trial [72]. | No | Not applicable | Mixed | Not discussed |
| **32** | A care coordinator screening strategy to address health harming legal needs [73]. | No | Not applicable | Mixed | Not discussed |
| **33** | Evidence that collaborative action between local health departments and nonprofit hospitals helps foster healthy behaviors in communities: a multilevel study [74]. | No | Not applicable | Community Partnerships | Not discussed |
| **34** | Hospital Partnerships for Population Health: A Systematic Review of the Literature [23]. | No | Not applicable | Community Partnerships | Not discussed |
| **35** | Medicaid Investments to Address Social Needs in Oregon and California [75]. | No | Not applicable | Mixed | Not discussed |
| **36** | Enabling Services Improve Access to Care, Preventive Services, And Satisfaction Among Health Center Patients [76]. | Yes | Not specified | Mixed | Not discussed |
| **37** | A Social-Return-On-Investment Analysis of Bon Secours Hospital's 'Housing for Health' Affordable Housing Program [77]. | Yes | Reduced neighborhood crime, increased safety, improved community well-being, reduced homelessness, increased housing stability, increased property values in the surrounding neighborhoods, reduced loneliness among seniors, and reduced vacant residential properties in the neighborhood | Internal capacity | Organizational challenges (limited documentation and reporting) |
| **38** | Embedding Social Workers in Veterans Health Administration Primary Care Teams Reduces Emergency Department Visits [78]. | No | Not applicable | Internal capacity | Not discussed |
| **39** | Project Nurture Integrates Care and Services to Improve Outcomes for Opioid-Dependent Mothers and Their Children [79]. | No | Not applicable | Mixed | Implementation challenges |
| **40** | Addressing the Poverty Barrier in Collaborative Care for Adults Experiencing Homelessness: A Case-Based Report [80]. | Yes | Not specified | Internal capacity | Limited best practice and research evidence (Inability to explore systemic impact of the program) |
| **41** | Interprofessional, multitiered daily rounding management in a high-acuity hospital [81]. | No | Not applicable | Internal capacity | Limited potential for replicability (Complex patient demographics) |
| **42** | Indianapolis Provider's Use of Wraparound Services Associated with Reduced Hospitalizations and Emergency Department Visits [82]. | No | Not applicable | Internal capacity | Limited best-practice and research evidence (Limitation in cost savings assessment) |
| **43** | Reasons for Social Work Referrals in an Urban Safety-Net Population: A Natural Language Processing and Market Basket Analysis Approach [83]. | No | Not applicable | Internal capacity | Organizational challenges (Lack of training and skill set in physicians to address social needs) |
| **44** | The Impact of Food Insecurity Screenings and Community Food Resource Referrals for Patients with Type 2 Diabetes [84]. | No | Not applicable | Mixed | Implementation challenges (Barriers to resources access) |
| **45** | The implementation cost of a safety‐net hospital program addressing social needs in Atlanta [85]. | Yes | Greater equity due to:  -Better access to prescribed medications,  -Better access and continuity of care  -Greater ability to keep appointments  -Greater ability to receive treatment at home  -Increased use of community services | Internal capacity | Limited potential for replicability (Complex patient demographics) |
| **46** | Development of a homelessness risk screening tool for emergency department patients [86]. | No | Not applicable | Internal capacity | Limited potential for replicability (Complex patient demographics) |
| **47** | An embedded multiple case study: using CFIR to map clinical food security screening constructs for the development of primary care practice guidelines [87]. | No | Not applicable | Mixed | Implementation challenges |
| **48** | Conceptualizing the effective mechanisms of a social needs case management program shown to reduce hospital use: a qualitative study [88]. | No | Not applicable | Mixed | Limited best-practice and research evidence (Lack of evidence about the mechanisms by which interventions benefit patients) |
| **49** | Social Needs Screening and Referral Program at a Large US Public Hospital System, 2017 [89]. | No | Not applicable | Internal capacity | Implementation challenges |
| **50** | A Nurse-Led Intervention to Address Food Insecurity in Chicago [90]. | No | Not applicable | Internal capacity | Insufficient community-level data to describe the magnitude of social need |
| **51** | Training Student Volunteers as "Community Resource Navigators" to Integrate Health and Social Care in Primary Care [91]. | No | Not applicable | Mixed | Organizational challenges (Training Limitations for Volunteer Models) |
| **52** | An In-Clinic Food Pharmacy Addresses Very Low Food Security [92]. | No | Not applicable | Internal capacity | Implementation challenges (Barriers to resources access) |
| **53** | Evidence based processes to prevent readmissions: more is better, a ten-site observational study [93]. | No | Not applicable | Internal capacity | Not discussed |
| **54** | Unmet Social Needs and No-Show Visits in Primary Care in a US Northeastern Urban Health System, 2018–2019 [94]. | No | Not applicable | Internal capacity | Not discussed |
| **55** | Cooling The Hot Spots Where Child Hospitalization Rates Are High: A Neighborhood Approach to Population Health [95]. | Yes | Disparities in hospitalization rates between different neighborhoods | Internal capacity | Not discussed |
| **56** | Quantifying Health Systems' Investment in Social Determinants of Health, By Sector, 2017–19 [96]. | No | Not applicable | Mixed | Implementation challenges (Lack of direct investment) |
| **57** | Prescribing Housing: A Scoping Review of Health System Efforts to Address Housing as a Social Determinant of Health [34]. | No | Not applicable | Mixed | Not discussed |
| **58** | The Baltimore Community-Based Organizations Neighborhood Network: Enhancing Capacity Together (CONNECT) Cluster RCT [97]. | No | Not applicable | Mixed | Not discussed |
| **59** | Effect of Community Health Worker Support on Clinical Outcomes of Low-Income Patients Across Primary Care Facilities: A Randomized Clinical Trial [98]. | No | Not applicable | Internal capacity | Organizational challenges (Lack of shared background with the disadvantaged patients) |
| **60** | Food for Thought: A Randomized Trial of Food Insecurity Screening in the Emergency Department [99]. | No | Not applicable | Internal capacity | Organizational challenges (Inability to verify self-reported data) |
| **61** | Effect of a Peer-Led Behavioral Intervention for Emergency Department Patients at High Risk of Fatal Opioid Overdose: A Randomized Clinical Trial [100]. | No | Not applicable | Mixed | Not discussed |
| **62** | Patient plus partner trial: A randomized controlled trial of 2 interventions to improve outcomes after an initial implantable cardioverter-defibrillator [101]. | No | Not applicable | Internal capacity | Not discussed |
| **63** | Illustrating the value of social work: results of an open pilot trial of the psychosocial acuity scale in a large urban pediatric hospital [102]. | No | Not applicable | Internal capacity | Organizational challenges (Limited documentation and reporting) |
| **64** | Using Publicly Available Data to Understand the Opioid Overdose Epidemic: Geospatial Distribution of Discarded Needles in Boston, Massachusetts [103]. | No | Not applicable | Internal capacity | Not discussed |
| **65** | Predictors for Poor Linkage to Care Among Hospitalized Persons Living with HIV and Co-Occurring Substance Use Disorder [104]. | No | Not applicable | Internal capacity | Not discussed |
| **66** | Enhanced care planning and clinical-community linkages versus usual care to address basic needs of patients with multiple chronic conditions: a clinician-level randomized controlled trial [105]. | No | Not applicable | Internal capacity | Not discussed |
| **67** | Social Risks Among Primary Care Patients in a Large Urban Health System [27]. | No | Not applicable | Internal capacity | Not discussed |
| **68** | Relationship of Neighborhood Deprivation and Outcomes of a Comprehensive ST-Segment-Elevation Myocardial Infarction Protocol [106]. | No | Not applicable | Internal capacity | Not discussed |
| **69** | Enhancing Reach Out and Read with a Video and Text Messages: A Randomized Trial in a Low-Income Predominantly Latino Sample [107]. | No | Not applicable | Internal capacity | Implementation challenges (Declining participation in the intervention) |
| **70** | Social Needs Screening in Hospitalized Pediatric Patients: A Randomized Controlled Trial [108]. | No | Not applicable | Mixed | Implementation challenges (Declining participation in the intervention) |
